# Pneumococcal Serotype-Specific Antibodies Persist through Early Childhood after Infant Immunization: Follow-Up from a Randomized Controlled Trial

**DOI:** 10.1371/journal.pone.0091413

**Published:** 2014-03-11

**Authors:** Johannes Trück, Matthew D. Snape, Florencia Tatangeli, Merryn Voysey, Ly-Mee Yu, Saul N. Faust, Paul T. Heath, Adam Finn, Andrew J. Pollard

**Affiliations:** 1 Oxford Vaccine Group, Department of Paediatrics, University of Oxford and the NIHR Oxford Biomedical Research Centre, Oxford, United Kingdom; 2 Centre for Statistics in Medicine, University of Oxford, Oxford, United Kingdom; 3 NIHR Wellcome Trust Clinical Research Facility and NIHR Respiratory Biomedical Research Unit, University Hospital Southampton NHS Foundations Trust and Faculty of Medicine and Institute for Life Sciences, University of Southampton, Southampton, United Kingdom; 4 St. George’s Vaccine Institute, St. George’s, University of London, London, United Kingdom; 5 Bristol Children’s Vaccine Centre, University Hospitals Bristol NHS Foundation Trust and University of Bristol, Bristol, United Kingdom; The George Washington University Medical Center, United States of America

## Abstract

**Background:**

In a previous UK multi-center randomized study 278 children received three doses of 7-valent (PCV-7) or 13-valent (PCV-13) pneumococcal conjugate vaccine at 2, 4 and 12 months of age. At 13 months of age, most of these children had pneumococcal serotype-specific IgG concentrations ≥0.35 µg/ml and opsonophagocytic assay (OPA) titers ≥8.

**Methods:**

Children who had participated in the original study were enrolled again at 3.5 years of age. Persistence of immunity following infant immunization with either PCV-7 or PCV-13 and the immune response to a PCV-13 booster at pre-school age were investigated.

**Results:**

In total, 108 children were followed-up to the age of 3.5 years and received a PCV-13 booster at this age. At least 76% of children who received PCV-7 or PCV-13 in infancy retained serotype-specific IgG concentrations ≥0.35 µg/ml against each of 5/7 shared serotypes. For serotypes 4 and 18C, persistence was lower at 22–42%. At least 71% of PCV-13 group participants had IgG concentrations ≥0.35 µg/ml against each of 4/6 of the additional PCV-13 serotypes; for serotypes 1 and 3 this proportion was 45% and 52%. In the PCV-7 group these percentages were significantly lower for serotypes 1, 5 and 7F. A pre-school PCV-13 booster was highly immunogenic and resulted in low rates of local and systemic adverse effects.

**Conclusion:**

Despite some decline in antibody from 13 months of age, these data suggest that a majority of pre-school children maintain protective serotype-specific antibody concentrations following conjugate vaccination at 2, 4 and 12 months of age.

**Trial Registration:**

ClinicalTrials.gov NCT01095471

## Introduction

The encapsulated bacterium *Streptococcus pneumoniae* (pneumococcus) is responsible for a considerable burden of disease in young children, both invasive disease such as meningitis, as well as mucosal infection such as otitis media and pneumonia. Worldwide more than 14 million episodes of serious pneumococcal disease and about 800,000 deaths in children under the age of five years occur annually [1]–[3].

Healthy pre-school children frequently carry pneumococci in the nasopharynx [4], [5]. Young children have the highest rates of pneumococcal colonization and are therefore thought to be the main transmitters of pneumococci throughout the population including to other young children and the elderly [6], which may result in disease in these populations. Maintaining adequate (functional) antibody levels in pre-school children is the key to sustained direct protection of these vaccinated individuals, as well as blocking transmission of the organism to achieve herd protection. Both are presumed to be mediated by antibody. Pneumococcal conjugate vaccines (PCVs) are highly effective in preventing carriage of the serotypes contained in the vaccine and thus disease [7]. Previous immunogenicity studies showed that with the currently used infant immunization schedules, immunity to pneumococcal serotypes wanes rapidly during the 2^nd^ year of life [8], [9].

In a previously reported phase II parallel-group, randomized, active-controlled, double-blind, multicenter trial [10], 278 children received either PCV-7 or PCV-13 at 2, 4 and 12 months of age along with routine immunizations for the United Kingdom. In this follow-on study, we evaluated the persistence of serotype-specific antibody at 3.5 years of age in individuals who had been enrolled into the earlier trial. In addition, we investigated the immunogenicity and reactogenicity of a PCV-13 vaccine booster at this age, comparing individuals previously primed with PCV-7 or PCV-13. The PCV-13 booster was studied at an age at which UK children routinely receive their pre-school immunizations as a possibility to enhance and maintain antibody levels in children under the age of five and to assess immunological questions relating to how PCV-7 vaccinated individuals respond to the 6 additional serotypes only contained in PCV-13.

## Methods

### Participants, Recruitment and Design

This follow-on, multi-center, open label, phase IV clinical trial was conducted from May to December 2010 in four sites (Oxford, London, Bristol, Southampton) across the United Kingdom. Five sites, enrolling 66 participants in the original study, did not take part in this follow-on study. The protocol for this trial and supporting CONSORT checklist are available as supporting information; see Checklist S1 and Protocol S1. Ethical approval was obtained from the Oxfordshire Research Ethics Committee C (Reference Number 10/H0606/09) and the study was registered on Clinicaltrials.gov (registration number: NCT01095471). Potential study participants were those who had completed the initial infant trial (6096A1; Clinicaltrials.gov registration number: NCT00384059), were healthy and 39–46 months of age. Exclusion criteria were similar to the initial study [10] and included previous anaphylactic reactions to any vaccine or vaccine-related component, bleeding diathesis, culture proven sepsis with *S. pneumoniae*, any serious or chronic ill health (including immunodeficiency), receipt of blood products, participation in another investigational study, and the receipt of further doses of pneumococcal vaccines since the initial study. Written informed consent was taken by appropriately trained medical or nursing members of the research team. The parent/guardian and the person who presented and obtained consent had to personally date and sign the latest approved version of the consent form before any study-specific procedures were performed, which was then retained at the study site. This procedure of taking and documenting consent was approved by the Oxfordshire Research Ethics Committee (Reference Number 10/H0606/9).

At visit 1, participants had a blood sample taken, followed by receipt of a dose of Prevenar 13 (PCV-13; Pfizer Inc., Pearl River, USA). A second blood sample was drawn at visit 2 (28 to 42 days after visit 1), together with doses of the routine pre-school immunizations (second dose of MMR vaccine and DTaP/IPV pre-school booster), if not already received. Laboratory staff remained blinded to original trial vaccine group allocation.

### Study Objectives

The primary objective of this study was to determine the proportion of participants immunized with PCV-13 at 2, 4 and 12 months of age, who had serotype-specific immunoglobulin G (IgG) concentrations ≥0.35 µg/ml for PCV-13 serotypes at pre-school age. The secondary objectives were to determine the proportion of participants with pre-booster serotype-specific IgG ≥0.35 µg/ml for PCV-13 serotypes in the PCV-7 group; the proportion of participants with opsonophagocytic assay (OPA) titers ≥8 for PCV-13 serotypes in both groups; and the immunogenicity as well as the reactogenicity of a booster dose of PCV-13 given at approximately 3.5 years of age.

### Vaccines and Interventions

PCV-13 is formulated in a similar manner to PCV-7 containing serotypes 4, 6B, 9V, 14, 18C, 19F, 23F and includes the additional serotypes 1, 3, 5, 6A, 7F, and 19A [11]. PCV-13 was administered to all participants intramuscularly into the deltoid muscle during visit 1, which took place at the participants’ homes by trained members of the research team. Routine pre-school booster immunizations were also administered to those who required them following the UK immunization program at visit 2. All vaccines were presented in prefilled syringes.

### Laboratory Measurements

Serum was separated within 24 hours of sampling and stored at −80°C. In both groups, PCV-13 serotype-specific IgG antibody concentrations were tested at Pfizer Vaccine Research, Pearl River, USA as previously described [10]. OPA titers against PCV-13 serotypes were calculated as the interpolated reciprocal dilution of test serum that causes a 50% reduction of bacteria surviving the opsonophagocytic reaction incubation. A titer of 4 was allocated to sera with undetectable OPAs, the titer of 8 being the lowest positive result. Serotype-specific antibody concentrations of ≥0.35 µg/ml and OPA titers ≥8 were used as putative correlates of immunity against serotype-specific IPD [1], [12].

### Statistical Analysis

Data were log_10_-transformed prior to analysis and results of group comparisons on log scales were presented as geometric means in each group, with 95% confidence interval and associated p-value from independent samples t-tests for the group comparison. Binary variables were presented as counts and proportions or percentages within each group, with 95% CI calculated using the binomial exact method and comparisons performed using chi-square tests. All statistical tests were 2-sided and p-values less than 0.05 were considered significant. For each participant the number of serotypes above threshold was calculated and standardized to 13 serotypes (to account for a small number of participants with some missing serotypes) by dividing the number of serotypes above threshold by the number of serotypes with available data for that participant. Participants were analyzed according to the group to which they had been randomized in the initial study. Statistical analysis were performed using SAS version 9.2 (SAS Institute Inc., Cary, NC, USA) and R [13].

## Results

**Figure 1:**
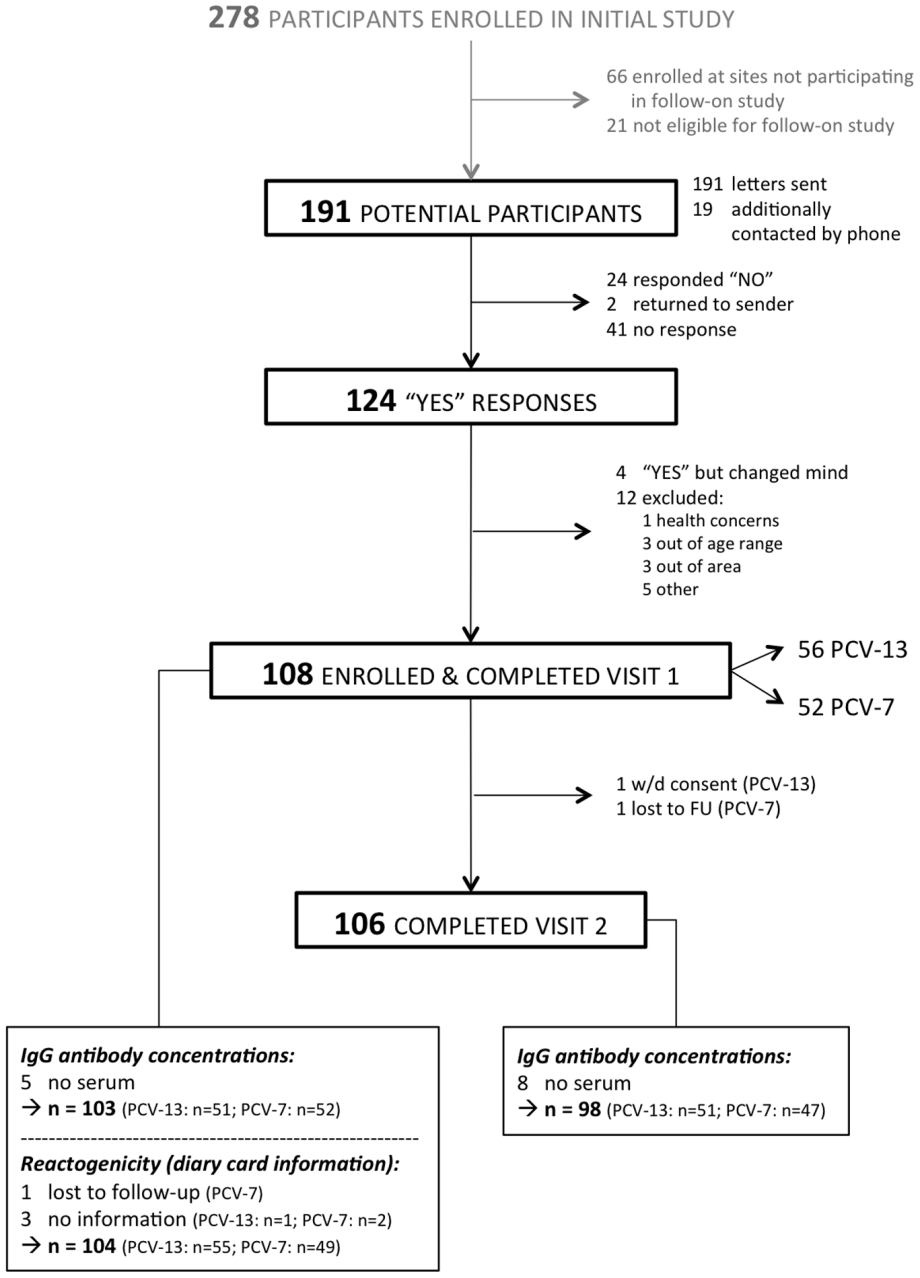
Flow chart of participants through the study.

Out of 278 participants enrolled in the original study, 191 were eligible for the follow-on study. Of these, 108 children were enrolled into the follow-on study and received the booster vaccine. The parents of one child withdrew consent before visit 2 and 1 child was lost to follow-up, resulting in 106 participants who completed visit 2 (Figure 1). The age, gender, time since last pneumococcal conjugate vaccination and the time interval between visit 1 and visit 2 were similar in both groups (Table 1).

**Table 1:** Summary of study participants’ characteristics.

	*PCV-13 (N = 56)*	*PCV-7 (N = 52)*
Proportion of female study participants	28 (50%)	28 (54%)
Proportion of White Caucasian study participants	54 (96%)	48 (92%)
Mean age at Visit 1 in years (range)	3.50 (3.16–4.09)	3.51 (3.15–4.04)
Mean time since 12-month booster to Visit 1 in years (range)	2.49 (2.13–3.07)	2.51 (2.15–2.97)
Mean time interval between Visit 1 - Visit 2 in days (range)	34.9 (26.5–56.5)	35.0 (27.6–42.6)

### Antibody Persistence to Pre-school Years

Approximately 2.5 years after completion of a 2+1 immunization schedule with PCV-13, most children had serotype-specific IgG concentrations ≥0.35 µg/ml, for the majority of serotypes against which they had been immunized (median number of serotypes with IgG above threshold: 10). This was also true for children immunized with PCV-7 as the primary vaccine (median number of serotypes with IgG above threshold: 9; Figure 2). Before the pre-school booster ≥76% of participants had IgG concentrations ≥0.35 µg/ml for 5 of the 7 shared serotypes, the exceptions being serotypes 4 and 18C (Table 2 and Figure 3). A significantly higher proportion of participants had IgG concentrations ≥0.35 µg/ml for serotype 4 in PCV-7 recipients compared with participants in the PCV-13 group (Figure 3). For the 6 additional PCV-13 serotypes, IgG concentrations ≥0.35 µg/ml were present in almost all participants in both groups for serotypes 6A and 19A (and 83 and 96% for serotype 5 in the PCV-7 and PCV-13 groups, respectively). Between the groups the proportions of participants with IgG concentrations ≥0.35 µg/ml were significantly higher in PCV-13 than PCV-7 recipients for serotypes 1, 5 and 7F (Table 2 and Figure 3).

**Figure 2:**
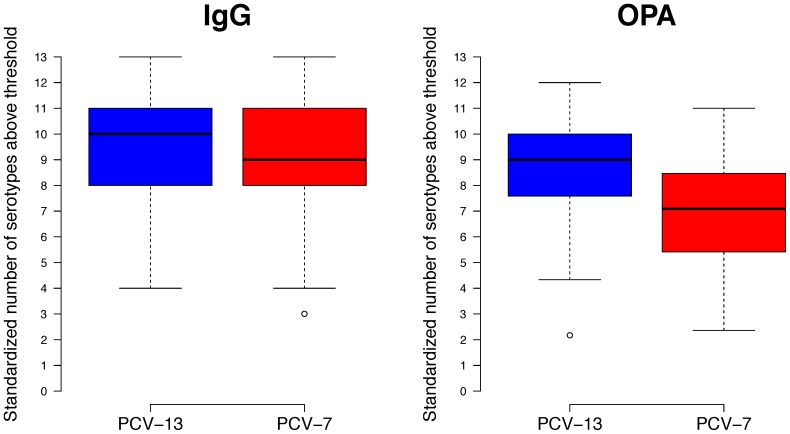
Standardized number of serotypes above thresholds. Box plots of standardized number of serotypes above thresholds (IgG ≥0.35 µg/ml and OPA titers ≥8) at 3.5 years in each of the groups [standardized to 13 serotypes]. The median is shown as a line across the box with the box representing the lower and upper quartiles. Whiskers extend to the maximum or minimum values within 1.5 times the IQR above and below the 3^rd^ and 1^st^ quartile, respectively. Points outside this range are represented as dots.

**Figure 3:**
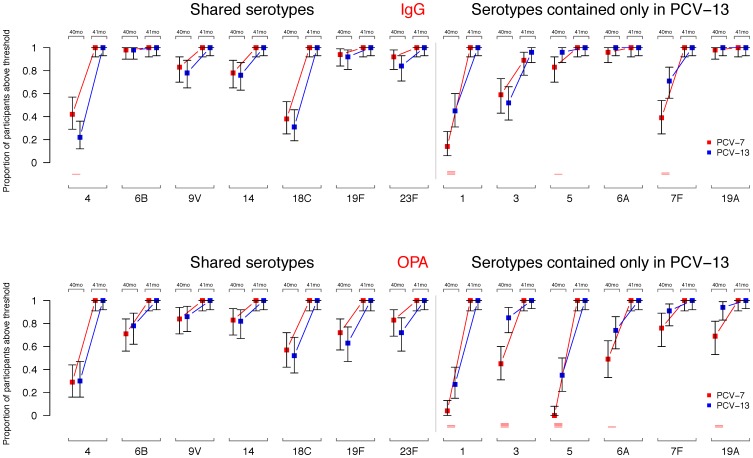
Proportion of participants with serotype-specific IgG concentrations and OPA titers above thresholds. Proportion of participants with serotype-specific IgG concentrations and OPA titers above a threshold of 0.35 µg/ml and 8, respectively, for each of the serotypes before (40 months, 40 mo) and after (41 months, 41 mo) the PCV-13 booster. Shown are mean values along with their 95% confidence intervals. Red lines are shown at the bottom if p-values from chi-square test of proportions between the groups were significant (p-values ≤0.05, ≤0.01, ≤0.001, and ≤0.0001 are represented by 1, 2, 3, and 4 lines).

**Table 2:** Proportion of participants with serotype-specific IgG concentrations ≥0.35 µg/ml.

	*––––– PREBOOSTER –40 months –––––*			*–– POSTBOOSTER –41 months ––*	
	*Proportion ≥0.35 µg/ml (95% CI)* *		*P value* **	*Proportion ≥0.35 µg/ml (95% CI)* *	
*Serotype*	*PCV-13*	*PCV-7*		*PCV-13*	*PCV-7*
4	0.22 (0.12, 0.36)	0.42 (0.29, 0.57)	**0.0284**	1.00 (0.93, 1.00)	1.00 (0.92, 1.00)
6B	0.98 (0.90, 1.00)	0.98 (0.90, 1.00)	0.9889	1.00 (0.93, 1.00)	1.00 (0.92, 1.00)
9V	0.78 (0.65, 0.89)	0.83 (0.70, 0.92)	0.5847	1.00 (0.93, 1.00)	1.00 (0.92, 1.00)
14	0.76 (0.63, 0.87)	0.78 (0.65, 0.89)	0.8127	1.00 (0.93, 1.00)	1.00 (0.92, 1.00)
18C	0.31 (0.19, 0.46)	0.38 (0.25, 0.53)	0.4506	1.00 (0.93, 1.00)	1.00 (0.92, 1.00)
19F	0.92 (0.81, 0.98)	0.94 (0.84, 0.99)	0.6759	1.00 (0.93, 1.00)	1.00 (0.92, 1.00)
23F	0.84 (0.71, 0.93)	0.92 (0.81, 0.98)	0.2061	1.00 (0.93, 1.00)	1.00 (0.92, 1.00)
6A†	1.00 (0.93, 1.00)	0.96 (0.87, 1.00)	0.1573	1.00 (0.93, 1.00)	1.00 (0.92, 1.00)
1†	0.45 (0.31, 0.60)	0.14 (0.06, 0.27)	**0.0006**	1.00 (0.93, 1.00)	1.00 (0.92, 1.00)
3†	0.52 (0.37, 0.66)	0.59 (0.43, 0.73)	0.5099	0.96 (0.87, 1.00)	0.89 (0.76, 0.96)
5†	0.96 (0.87, 1.00)	0.83 (0.70, 0.92)	**0.0279**	1.00 (0.93, 1.00)	1.00 (0.92, 1.00)
7F†	0.71 (0.56, 0.83)	0.39 (0.25, 0.54)	**0.0014**	1.00 (0.93, 1.00)	1.00 (0.92, 1.00)
19A†	1.00 (0.93, 1.00)	0.98 (0.90, 1.00)	0.3196	1.00 (0.93, 1.00)	1.00 (0.92, 1.00)

*Binomial exact confidence interval;

**P Value from chi-square test of proportions at 40 months, no comparisons were made at 41 months as almost all proportions were 1;

† Serotypes only present in PCV-13.

**Figure 4:**
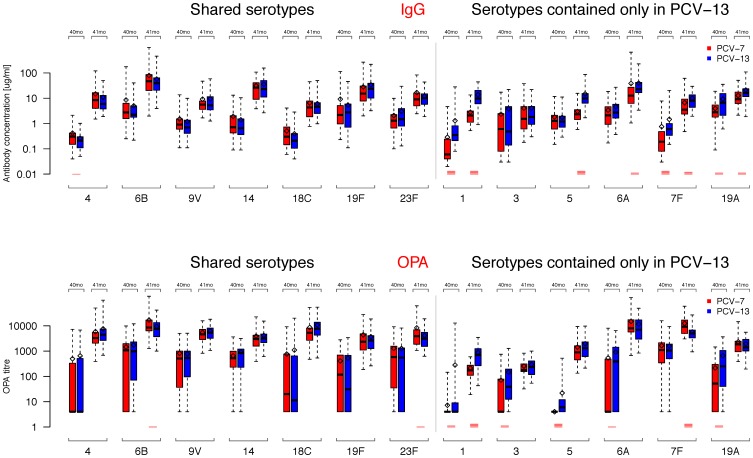
Serotype-specific IgG GMCs and OPA GMTs. Box plots of IgG GMCs and OPA GMTs for each of the serotypes before (40 months, 40 mo) and after (41 months, 41 mo) the PCV-13 booster. The median is shown as a line across the box with the box extending to the lower and upper quartiles, respectively. The whiskers extend to the maximum or minimum values within 1.5 times the IQR above and below the 3^rd^ and 1^st^ quartile, respectively. Points outside this range are represented as dots. Means are shown as a diamonds and t-test were performed for each serotype at each time point between the groups with p-values being represented as red lines if significant (1, 2, 3, and 4 lines if p≤0.05, ≤0.01, ≤0.001, and ≤0.0001, respectively).

Serotype specific IgG geometric mean concentrations (GMCs) were significantly higher in PCV-13 than PCV-7 recipients for serotypes 1, 7F and 19A (Figure 4 and Table 3), but did not significantly differ for the other three serotypes contained only in PCV-13. For serotypes included in both vaccines, only serotype 4 was significantly different between the groups (slightly higher in the PCV-7 group).

**Table 3:** Serotype-specific geometric mean concentrations before and after the booster at 40 and 41 months of age with t-tests.

	*––––– PREBOOSTER –40 months –––––*			*––––– POSTBOOSTER –41 months –––––*		
	*GMC (95% CI)*		*P value* **	*GMC (95% CI)*		*P value* **
*Serotype*	*PCV-13*	*PCV-7*		*PCV-13*	*PCV-7*	
4	0.20 (0.16, 0.24)	0.28 (0.22, 0.36)	**0.0267**	7.37 (5.80, 9.37)	8.72 (6.48, 11.73)	0.3744
6B	2.79 (2.06, 3.78)	3.28 (2.35, 4.57)	0.4779	37.82 (29.57, 48.37)	40.25 (28.78, 56.28)	0.7616
9V	0.76 (0.59, 0.98)	0.91 (0.69, 1.19)	0.3532	6.43 (5.06, 8.16)	6.23 (4.89, 7.94)	0.8546
14	0.75 (0.54, 1.02)	0.91 (0.65, 1.28)	0.3800	22.51 (17.00, 29.82)	21.51 (16.41, 28.18)	0.8145
18C	0.22 (0.16, 0.30)	0.31 (0.23, 0.41)	0.1051	4.33 (3.48, 5.41)	4.25 (3.25, 5.55)	0.9054
19F	2.30 (1.55, 3.42)	2.65 (1.78, 3.94)	0.6135	22.18 (17.05, 28.87)	15.29 (11.16, 20.96)	0.0701
23F	1.48 (1.05, 2.10)	1.31 (0.99, 1.72)	0.5646	9.10 (7.33, 11.32)	10.25 (7.84, 13.39)	0.4897
6A†	3.04 (2.34, 3.94)	2.14 (1.63, 2.83)	0.0684	26.81 (21.01, 34.20)	13.55 (9.27, 19.80)	**0.0032**
1†	0.45 (0.32, 0.62)	0.10 (0.07, 0.15)	**<.0001**	10.16 (8.04, 12.84)	2.03 (1.61, 2.56)	**<.0001**
3†	0.71 (0.40, 1.28)	0.56 (0.32, 0.99)	0.5552	2.18 (1.59, 2.98)	1.71 (1.16, 2.52)	0.3357
5†	1.22 (0.97, 1.53)	1.11 (0.84, 1.48)	0.6168	10.46 (8.34, 13.13)	2.28 (1.84, 2.81)	**<.0001**
7F†	0.67 (0.51, 0.90)	0.25 (0.17, 0.37)	**<.0001**	7.82 (6.38, 9.59)	4.32 (3.33, 5.61)	**0.0004**
19A†	5.34 (3.84, 7.42)	2.75 (2.11, 3.58)	**0.0020**	15.70 (12.62, 19.54)	9.49 (7.54, 11.94)	**0.0019**

**P value from independent samples t-test using Satterthwaites method for unequal variances where appropriate.

† Serotypes only present in PCV-13 vaccine.

Serotype-specific functional antibodies were also assessed by OPA. Similar to the IgG data, a high proportion of participants had OPA titers ≥8 for most of the serotypes in the PCV-13 group (median number of serotypes with OPA above threshold: 9), which was significantly lower for the PCV-7 group with a median of 7.1 (Figure 2, p<0.001). Before the PCV-13 booster, proportions of participants with OPA titers ≥8 for the 7 shared serotypes were high for serotypes 9V and 14 (82–86%), 63–83% for 6B, 19F and 23F and lower for serotypes 18C (52–57%) and 4 (29–30%) (Figure 3). For the 6 additional serotypes OPA titers ≥8 were observed in 85–94% of PCV-13 group participants for serotypes 3, 7F and 19A and 74% for 6A, but were lower for serotypes 1 (27%) and 5 (35%) (Figure 3). In the PCV-7 group, 69–74% of participants had OPA titers ≥8 for serotypes 7F and 19A, 45–49% for 3 and 6A and very few participants had detectable OPA titers against serotypes 1 and 5 (Table 4). OPA geometric mean titers (GMTs) at 40 months of age before the PCV-13 booster were similar between the groups for the shared serotypes. As for the PCV-13 serotypes, significant differences were observed for serotypes 1, 3, 5, 6A and 19A but not for serotype 7F, for which there were very high OPA titers in both groups (Table 5 and Figure 4).

**Table 4:** Proportion of participants with serotype-specific OPA titers ≥8.

	*––––– PREBOOSTER –40 months –––––*			*–– POSTBOOSTER –41 months ––*	
	*Proportion OPA ≥8 (95% CI)* *		*P value* **	*Proportion OPA ≥8 (95% CI)* *	
*Serotype*	*PCV-13*	*PCV-7*		*PCV-13*	*PCV-7*
4	0.30 (0.16, 0.47)	0.29 (0.16, 0.44)	0.9336	1.00 (0.92, 1.00)	1.00 (0.91, 1.00)
6B	0.78 (0.62, 0.89)	0.71 (0.56, 0.84)	0.5021	1.00 (0.92, 1.00)	1.00 (0.91, 1.00)
9V	0.86 (0.73, 0.95)	0.84 (0.71, 0.94)	0.7977	1.00 (0.92, 1.00)	1.00 (0.91, 1.00)
14	0.82 (0.67, 0.92)	0.83 (0.70, 0.93)	0.8481	1.00 (0.92, 1.00)	1.00 (0.91, 1.00)
18C	0.52 (0.37, 0.68)	0.57 (0.42, 0.72)	0.6201	1.00 (0.92, 1.00)	1.00 (0.91, 1.00)
19F	0.63 (0.47, 0.77)	0.72 (0.57, 0.84)	0.3681	1.00 (0.91, 1.00)	1.00 (0.91, 1.00)
23F	0.72 (0.56, 0.85)	0.83 (0.69, 0.92)	0.2350	1.00 (0.92, 1.00)	1.00 (0.91, 1.00)
6A†	0.74 (0.58, 0.86)	0.49 (0.33, 0.65)	**0.0192**	1.00 (0.92, 1.00)	1.00 (0.91, 1.00)
1†	0.27 (0.15, 0.42)	0.04 (0.00, 0.13)	**0.0013**	1.00 (0.92, 1.00)	1.00 (0.91, 1.00)
3†	0.85 (0.72, 0.94)	0.45 (0.31, 0.60)	**<.0001**	1.00 (0.93, 1.00)	1.00 (0.91, 1.00)
5†	0.35 (0.21, 0.50)	0.0 (0.0, 0.08)	**<.0001**	1.00 (0.92, 1.00)	1.00 (0.91, 1.00)
7F†	0.91 (0.78, 0.97)	0.76 (0.60, 0.89)	0.0712	1.00 (0.92, 1.00)	1.00 (0.91, 1.00)
19A†	0.94 (0.83, 0.99)	0.69 (0.53, 0.82)	**0.0019**	1.00 (0.93, 1.00)	1.00 (0.91, 1.00)

*Binomial exact confidence interval;

**P Value from chi-square test of proportions at 40 months, no comparisons were made at 41 months as all proportions were 1;

† Serotypes only present in PCV-13.

**Table 5:** Serotype-specific geometric mean OPA titers before and after the booster at 40 and 41 months of age with t-tests.

	*––––– PREBOOSTER –40 months –––––*			*––––– POSTBOOSTER –41 months –––––*		
	*GMT (95% CI)*		*P value* **	*GMT (95% CI)*		*P value* **
*Serotype*	*PCV-13*	*PCV-7*		*PCV-13*	*PCV-7*	
4	22.98 (9.13, 57.82)	19.47 (8.99, 42.19)	0.7797	4453 (3407, 5821)	3320 (2373, 4644)	0.1671
6B	338.6 (145.3, 789.3)	241.1 (103.7, 560.5)	0.5690	6605 (5013, 8702)	9962 (7313, 13571)	**0.0478**
9V	240.5 (126.2, 458.4)	229.7 (118.7, 444.3)	0.9200	5069 (4149, 6193)	4433 (3547, 5540)	0.3675
14	337.3 (171.7, 662.5)	264.3 (147.8, 472.6)	0.5808	3179 (2551, 3961)	2886 (2244, 3711)	0.5593
18C	50.37 (21.32, 119.0)	56.72 (25.52, 126.1)	0.8386	7530 (5780, 9809)	5119 (3713, 7058)	0.0624
19F	59.72 (27.45, 130.0)	73.30 (37.42, 143.6)	0.6877	2249 (1612, 3140)	2496 (1743, 3576)	0.6682
23F	179.4 (76.38, 421.3)	291.7 (142.5, 597.2)	0.3792	2972 (2316, 3815)	4466 (3236, 6162)	**0.0452**
6A†	172.4 (78.84, 376.8)	45.43 (19.84, 104.1)	**0.0204**	7023 (5072, 9724)	8814 (6351, 12232)	0.3251
1†	8.02 (5.34, 12.04)	4.45 (3.80, 5.21)	**0.0086**	588.2 (435.8, 793.9)	175.2 (140.6, 218.3)	**<.0001**
3†	46.37 (28.61, 75.13)	16.16 (9.86, 26.50)	**0.0028**	227.1 (183.3, 281.4)	209.9 (171.3, 257.2)	0.5965
5†	8.00 (5.93, 10.79)	4.00 (4.00, 4.00)	**<.0001**	1296 (976.5, 1719)	921.6 (683.8, 1242)	0.0978
7F†	677.2 (380.0, 1207)	354.3 (148.7, 844.5)	0.2134	4696 (3866, 5704)	9313 (7287, 11902)	**<.0001**
19A†	187.0 (105.7, 330.8)	50.25 (27.27, 92.60)	**0.0021**	1557 (1261, 1923)	1649 (1276, 2131)	0.7274

**P value from independent samples t-test using Satterthwaites method for unequal variances where appropriate.

† Serotypes only present in PCV-13 vaccine.

### Immunogenicity of the PCV-13 Pre-school Booster

IgG GMCs and OPA GMTs following the booster dose are shown in Table 3 and 5; these values were significantly higher than pre-booster for all serotypes (all p-values <0.00001, data not shown). All participants from both groups had IgG concentrations ≥0.35 µg/ml for all serotypes with the exception of serotype 3 (PCV-13 group: 96% and PCV-7 group: 89%; Figure 3). Post-booster antibody GMCs were significantly higher in the PCV-13 group for 5/6 of the additional serotypes only included in PCV-13, except for serotype 3 (Table 3 and Figure 4). Following the PCV-13 booster, all participants had OPA titers ≥8 for all serotypes. Significant differences in post-booster GMTs were observed for serotypes 6B and 23F from the shared serotypes (higher in the PCV-7 group) and for serotypes 1 (higher in the PCV-13 group) and 7F (higher in the PCV-7 group) – Table 5 and Figure 4.

### Reactogenicity

No serious adverse events were recorded following the PCV-13 booster at 3.5 years of age. Local reactions such as redness, hardness and swelling were either absent or mild in most cases. However, moderate or severe tenderness was experienced by 18% of all participants (Table 6). Decreased appetite and irritability were recorded in 18% and 41% of participants, respectively, which were moderate or severe in 4% and 13% of children. Low-grade fever (38–39°C) was noted in 3% of the participants and none of the children had a temperature >39°C (Table 6). The reactogenicity profile of the PCV-13 booster was similar regardless of whether participants had previously been immunized with PCV-7 or PCV-13.

**Table 6:** Local and systemic reactions following the pre-school PCV-13 booster at 40 months of age.

		*PCV-13* *(N = 55)*		*PCV-7 (N = 49;* *48 for T°)*			*Both groups (N = 104;* *103 for T°)*	
*Reaction*	*Level*	*N*	*Proportion*	*N*	*Proportion*	*P value* **	*N*	*Proportion*
**Redness**	Any	26	47%	20	41%	0.5566	46	44%
	Severe ≥5 cm	3	6%	1	2%		4	4%
**Swelling**	Any	8	15%	14	29%	0.0959	22	21%
	Severe ≥5 cm	1	2%	1	2%		2	2%
**Hardness**	Any	10	18%	16	33%	0.1136	26	25%
	Severe ≥5 cm	0	0%	0	0%		0	0%
**Tenderness**	Any	32	58%	28	57%	1.0000	60	58%
	Discomfort during routine activities	9	16%	6	12%		15	14%
	Interfering with limb movement	2	4%	2	4%		4	4%
**Poor appetite**	Appetite as usual	45	82%	40	82%	0.2322	19	18%
	Eating less/no effect on normal activity	6	11%	9	18%		15	14%
	Interfering with normal activity	3	6%	0	0%		3	3%
	Not eating at all	1	2%	0	0%		1	1%
**Irritability**	Behavior as usual	27	49%	34	70%	0.1925	43	41%
	More irritable/no effect on normal activity	18	33%	11	22%		29	28%
	Interfering with normal activity	6	11%	2	4%		8	8%
	Preventing normal activity	4	7%	2	4%		6	6%
**Axillary** **Temperature**	<38.0°C	55	100%	45	94%	0.0978	100	97%
	38.0–38.4°C	0	0%	2	4%		2	2%
	38.5–38.9°C	0	0%	1	2%		1	1%

**Fisher’s Exact Test.

## Discussion

This is to our knowledge the first clinical trial investigating the persistence of total and opsonophagocytic serotype-specific antibodies up to the age of 3.5 years following a CRM_197_-based 7- or 13-valent PCV schedule. We have shown that sustained effectiveness of both direct and indirect effects can be anticipated on a population level if based on the currently accepted “protective thresholds”. For the PCV-7 serotypes, this finding is consistent with IPD effectiveness data demonstrating almost complete disappearance of vaccine serotypes over a period of approximately 5–7 years [14]–[16] so that, at the population level, these responses appear to be adequate for effective disease control.

Only 2 other studies looked at the persistence of pneumococcal antibody beyond the second year of life. Silfverdal *et al.* [17] assessed the immune response in children aged 36–46 months previously vaccinated with the 10-valent PCV (PCV-10) in a 2+1 or 3+1 schedule compared to an unvaccinated control group of children of the same age. Although the focus of their work was to demonstrate induction of memory by PCV-10, they report information on the baseline antibody GMC and OPA GMT, which were generally lower for the majority of the vaccine serotypes even in the group of children who were vaccinated in a 3+1 schedule compared with our study. In a further study by Madhi *et al.* [18] in South Africa, trial participants who had received 3 doses of PCV-9 at approximately 6, 11 and 16 weeks of age were followed to approximately 6 years and compared with unvaccinated controls for age. Efficacy against vaccine-type IPD remained high (78%) in HIV uninfected children, which was associated with a high proportion of participants (≥75%) maintaining IgG concentrations ≥0.35 µg/ml against 5/7 PCV-7 serotypes. Lower proportions of participants reached the threshold against serotypes 4 and 18C, which is similar to our study. No functional antibody measurements by OPA were performed in that study.

There are data available on persistence of antibody against other polysaccharide-encapsulated bacteria following immunization with glycoconjugate vaccines in infancy [6]. In a recent study, the proportion of individuals with functional antibodies against serogroup C meningococci six years after vaccine priming was low (12–16%) after 2 or 3 doses in infancy [19]. In contrast, persistence of antibodies after infant vaccination with *H. influenzae* type b conjugate was somewhat better with a high proportion of vaccinees (86–99%) having antibodies above the protective threshold at age 3–4 years [20], [21].

As shown in previous studies there is variation of immunogenicity and persistence between different pneumococcal serotypes. In the present study, we evaluated the persistence of total and functional antibody against all PCV-13 serotypes in children who had received PCV-13 and PCV-7 in a 2+1 schedule. Proportions of participants with serotype-specific IgG concentrations ≥0.35 µg/ml and absolute antibody concentrations were not different between the groups for the shared serotypes, with the exception of serotype 4 for which higher responses were found in the PCV-7 group at 40 months. Data from the original study showed significant differences between the GMCs from PCV-7 and PCV-13 recipients for serotype 4 at all study visits (5, 12, and 13 months) [10] suggesting that the lower persistence against serotype 4 in the PCV-13 group observed in this follow-on study is not a chance finding. The lower antibody levels seen for serotypes 4 and 18C may be acceptable in populations with high PCV uptake that establishes herd immunity, as shown by large decreases in invasive disease cases both in vaccinated and unvaccinated individuals [22]. In the original part of the study, OPA GMTs were relatively low for serotypes 4, 18C and 19F several months after vaccination at 12 months of age despite a good initial immune response [10] indicating that antibodies directed against these serotypes wane rapidly irrespective of the age at immunization and the type of vaccine given. The reason for this finding is unknown and may be partly explained with low carriage rates for serotype 4 and 18C whereas rates of colonization for serotype 19F seem to be similar or higher compared with other PCV-7 serotypes [24], [25].

Participants in both groups showed a very good immune response to all 7 shared serotypes following the pre-school booster with PCV-13 suggesting that PCV-7 and PCV-13 have comparable priming ability for the shared serotypes. Overall similar immunogenicity and persistence for these 7 serotypes induced by the two vaccines is consistent with early PCV-13 post-licensure data from the UK showing equivalent effectiveness against these serotypes [23].

Marked differences were present between the groups for the serotypes contained only in PCV-13 both before and after the pre-school booster. We found low proportions of participants with OPA ≥8 and lower GMTs in the PCV-7 group at baseline and highly significant differences in GMT between the groups for serotypes 1 and 5 (Figures 3 and 4). Both serotypes were very rarely found in carriage studies both before and after introduction of PCV-7 into the UK routine immunization schedule [7], [24], [25], which may account for the low antibody levels seen in the PCV-7 group until the pre-school age compared with the other serotypes contained only in PCV-13. The significance of lower antibody persistence for serotypes 1 and 5 is currently unknown although there is some evidence that herd immunity is rapidly accumulating for most of the 6 additional serotypes [23], which implies that transmission at least in part can be blocked. Following the pre-school booster, significant differences between the groups remained for serotype 1, but not for serotype 5, when measured by OPA suggesting that priming with serotype 1 is important (Table 5). This seems to be less critical for serotype 5 for which vaccine unprimed individuals mounted a very good immune response (when measured by OPA) suggesting that at least for this serotype age may be a more important factor than the number of previous vaccine doses in determining the amount of functional antibody generated, as previously shown for other polysaccharide-containing vaccines [19], [21]. Post-licensure surveillance data from the UK show that children aged <5 years vaccinated with PCV-7 are not protected against serotypes 1 and 5 [22], which is consistent with low functional antibody titers in our study in the PCV-7 group (Figure 4).

Serotypes 6A and 19A are usually referred to as “cross-reactive” serotypes because antibodies induced by serotypes 6B and 19F cross-react *in vitro* with those 2 serotypes, respectively. We have shown in this study that PCV-7 induces high total and functional antibodies against 6A [10], which also persist at high levels up to the pre-school years (Figures 3 and 4). There is some evidence from both the US and the UK that cross-reactive antibodies against serotype 6A are effective in preventing carriage and disease [7], [16], [22], [25], [26], although serotype 6C isolates were initially misclassified as 6A, and changes in IPD rates caused by 6A may also depend on other factors than vaccination [27]. A completely different picture can be seen for serotype 19A. Although less pronounced compared with serotype 6A, PCV-7 induces a good [10] and persistent immune response against serotype 19A in addition to potential natural acquisition of antibody through colonization (Figures 3 and 4). Although *in vitro* these cross-reactive antibodies appear to be functional, PCV-7 vaccinated children are, in fact, at increased risk of serotype 19A disease following widespread PCV-7 immunization, compared with the pre-vaccine era [16], [22], most likely as a result of an increase in 19A carriage rates [7], [25], [28]. There are several potential reasons for this discrepancy between the *in vitro* and *in vivo* data: (a) OPA measures functional antibodies using one particular laboratory strain, which may differ significantly from disease isolates; (b) higher antibody concentrations/titers may be necessary to protect against IPD compared with other serotypes, or antibody-mediated opsonophagocytosis may not be the main mechanism of killing for this serotype; early data from the UK, however, indicate that PCV-13 is highly effective against serotype 19A disease [29]; (c) there is a theoretical possibility that a small percentage of individuals who are not able to generate cross-reactive antibodies, along with increased carriage rates in PCV-7 vaccinated populations, may be responsible for replacement disease.

In the present study, antibody concentrations and functional OPA titers against serotypes 3 and 7F in the PCV-7 group increased over time irrespective of vaccination [10], so that GMC/GMT were relatively high at 40 months of age (Figure 4). Although the kinetics of the acquisition of these antibodies suggest that this increase in (functional) antibody is most likely a result of natural exposure, low carriage rates were found in young children both before [26] and after [7], [25] routine PCV-7 immunization. These *in vitro* functional antibodies don’t appear to be effective in preventing IPD caused by serotypes 3 and 7F as shown by post-licensure effectiveness studies [16], [22]. It is again unclear how these antibodies differ from those generated by PCV-13, for which early vaccine effectiveness against serotype 7F has been demonstrated [29]. Serotype 3 may behave differently from other serotypes both in terms of immunogenicity and vaccine efficacy [30], [31] and it is possible that higher thresholds are required to prevent disease caused by serotype 3 [29].

### Limitations and Strengths of the Study

There are several potential limitations to our study. This study only allows a snapshot of the antibody concentrations and OPA titers at a certain time point. Differences in serotype epidemiology due to forces related and unrelated to routine immunization mean that serology is likely to be different in other populations at different times. Nevertheless the children studied here, alongside routine use of PCV-7 in the general population, permit interpretation of our findings together with UK post-licensure surveillance data.

## Conclusion

We have shown that following PCV-13 immunization at 2, 4 and 12 months of age, antibody persistence against most serotypes can be expected until the age of at least 3.5 years, which is consistent with post-licensure IPD effectiveness and carriage data [14]–[16]. However, this might not be the case either in situations with lower vaccine coverage [32] or natural acquired (functional) antibodies as shown in the present study for serotypes 3, 7F and 19A. Accordingly, serological studies of this kind are important alongside population surveillance of invasive disease, carriage studies and information on vaccine coverage to obtain information on the relationships between vaccine responses and population-wide effectiveness.

A pre-school booster with PCV-13 is well tolerated with low rates of local and systemic side effects after priming with either PCV-7 or PCV-13.

## Supporting Information

Checklist S1 CONSORT Checklist
(DOC)

Protocol S1 Trial Protocol
(PDF)

## References

[1] WHO (2007) Pneumococcal conjugate vaccine for childhood immunization–WHO position paper. Wkly Epidemiol Rec 82: 93–104. 17380597

[2] O’Brien KL, Wolfson LJ, Watt JP, Henkle E, Deloria-Knoll M, et al (2009) Burden of disease caused by Streptococcus pneumoniae in children younger than 5 years: global estimates. Lancet 374: 893–902. 19748398 10.1016/S0140-6736(09)61204-6

[3] Scott JA (2007) The preventable burden of pneumococcal disease in the developing world. Vaccine 25: 2398–2405. 17028081 10.1016/j.vaccine.2006.09.008

[4] Sleeman KL, Daniels L, Gardiner M, Griffiths D, Deeks JJ, et al (2005) Acquisition of Streptococcus pneumoniae and Nonspecific Morbidity in Infants and Their Families. Pediatr Infect Dis J 24: 121–127. 15702039 10.1097/01.inf.0000151030.10159.b1

[5] Garcia-Rodriguez A (2002) Dynamics of nasopharyngeal colonization by potential respiratory pathogens. Journal of Antimicrobial Chemotherapy 50: 59–74. 10.1093/jac/dkf506 12556435

[6] Trück J, Pollard AJ (2010) Challenges in immunisation against bacterial infection in children. Early Hum Dev 86: 695–701. 20851537 10.1016/j.earlhumdev.2010.08.010

[7] Flasche S, Van Hoek AJ, Sheasby E, Waight P, Andrews N, et al (2011) Effect of pneumococcal conjugate vaccination on serotype-specific carriage and invasive disease in England: a cross-sectional study. PLoS Med 8: e1001017. 21483718 10.1371/journal.pmed.1001017 PMC3071372

[8] Ekström N, Ahman H, Verho J, Jokinen J, Väkeväinen M, et al (2005) Kinetics and avidity of antibodies evoked by heptavalent pneumococcal conjugate vaccines PncCRM and PncOMPC in the Finnish Otitis Media Vaccine Trial. Infect Immun 73: 369–377. 15618174 10.1128/IAI.73.1.369-377.2005 PMC538941

[9] Givon-Lavi N, Greenberg D, Dagan R (2010) Immunogenicity of alternative regimens of the conjugated 7-valent pneumococcal vaccine: a randomized controlled trial. Pediatr Infect Dis J 29: 756–762. 20661103 10.1097/INF.0b013e3181d99345

[10] Snape MD, Klinger CL, Daniels ED, John TM, Layton H, et al (2010) Immunogenicity and Reactogenicity of a 13-Valent-pneumococcal Conjugate Vaccine Administered at 2, 4, and 12 Months of Age. Pediatr Infect Dis J 29: e80–e90. 21155091 10.1097/inf.0b013e3181faa6be

[11] Duggan ST (2012) Pneumococcal polysaccharide conjugate vaccine (13-valent, adsorbed) [Prevenar 13(®)]: profile report. Paediatr Drugs 14: 67–69. 22149553 10.2165/11207010-000000000-00000

[12] Henckaerts I, Durant N, De Grave D, Schuerman L, Poolman J (2007) Validation of a routine opsonophagocytosis assay to predict invasive pneumococcal disease efficacy of conjugate vaccine in children. Vaccine 25: 2518–2527. 17034907 10.1016/j.vaccine.2006.09.029

[13] R Core Team (2013) R: A Language and Environment for Statistical Computing. R Foundation for Statistical Computing.

[14] Hsu KK, Shea KM, Stevenson AE, Pelton SI (2010) Changing serotypes causing childhood invasive pneumococcal disease: Massachusetts, 2001–2007. Pediatr Infect Dis J 29: 289–293. 19935447 10.1097/INF.0b013e3181c15471

[15] Hanage WP, Finkelstein JA, Huang SS, Pelton SI, Stevenson AE, et al (2010) Evidence that pneumococcal serotype replacement in Massachusetts following conjugate vaccination is now complete. Epidemics 2: 80–84. 21031138 10.1016/j.epidem.2010.03.005 PMC2963072

[16] Pilishvili T, Lexau C, Farley MM, Hadler J, Harrison LH, et al (2010) Sustained reductions in invasive pneumococcal disease in the era of conjugate vaccine. J Infect Dis 201: 32–41. 19947881 10.1086/648593

[17] Silfverdal SA, Skerlikova H, Zanova M, Papuchova D, Traskine M, et al (2011) Anamnestic Immune Response in 3- to 4-year-old Children Previously Immunized With 10-valent Pneumococcal Nontypeable Haemophilus influenzae Protein D Conjugate Vaccine as 2 Dose or 3 Dose Priming and a Booster Dose in the First Year of Life. Pediatr Infect Dis J 30: e155–e163. 21572373 10.1097/INF.0b013e31821feeb7

[18] Madhi SA, Adrian P, Kuwanda L, Jassat W, Jones S, et al (2007) Long-term immunogenicity and efficacy of a 9-valent conjugate pneumococcal vaccine in human immunodeficient virus infected and non-infected children in the absence of a booster dose of vaccine. Vaccine 25: 2451–2457. 17023095 10.1016/j.vaccine.2006.09.019

[19] Perrett KP, Winter AP, Kibwana E, Jin C, John TM, et al (2010) Antibody persistence after serogroup C meningococcal conjugate immunization of United Kingdom primary-school children in 1999–2000 and response to a booster: a phase 4 clinical trial. Clin Infect Dis 50: 1601–1610. 20459323 10.1086/652765

[20] Heath PT, Booy R, Azzopardi HJ, Slack MP, Bowen-Morris J, et al (2000) Antibody concentration and clinical protection after Hib conjugate vaccination in the United Kingdom. JAMA 284: 2334–2340. 11066183 10.1001/jama.284.18.2334

[21] Khatami A, Snape MD, John T, Westcar S, Klinger C, et al (2011) Persistence of immunity following a booster dose of Haemophilus influenzae type B-Meningococcal serogroup C glycoconjugate vaccine: follow-up of a randomized controlled trial. Pediatr Infect Dis J 30: 197–202. 20844459 10.1097/INF.0b013e3181f728fd

[22] Miller E, Andrews NJ, Waight PA, Slack MP, George RC (2011) Herd immunity and serotype replacement 4 years after seven-valent pneumococcal conjugate vaccination in England and Wales: an observational cohort study. Lancet Infect Dis 11: 760–768. 21621466 10.1016/S1473-3099(11)70090-1

[23] JCVI (2012) Minutes of meeting on Wednesday 30 May 2012. Joint committee on vaccination and immunisation (Pneumococcal sub-committee).

[24] Sleeman KL, Griffiths D, Shackley F, Diggle L, Gupta S, et al (2006) Capsular serotype-specific attack rates and duration of carriage of Streptococcus pneumoniae in a population of children. J Infect Dis 194: 682–688. 16897668 10.1086/505710

[25] Tocheva AS, Jefferies JM, Rubery H, Bennett J, Afimeke G, et al (2011) Declining serotype coverage of new pneumococcal conjugate vaccines relating to the carriage of Streptococcus pneumoniae in young children. Vaccine 29: 4400–4404. 21504773 10.1016/j.vaccine.2011.04.004

[26] Hussain M, Melegaro A, Pebody RG, George R, Edmunds WJ, et al (2005) A longitudinal household study of Streptococcus pneumoniae nasopharyngeal carriage in a UK setting. Epidemiol Infect 133: 891–898. 16181510 10.1017/S0950268805004012 PMC2870321

[27] Rudolph K, Bruce M, Bruden D, Zulz T, Wenger J, et al (2012) Epidemiology of pneumococcal serotype 6A and 6C among invasive and carriage isolates from Alaska, 1986–2009. Diagn Microbiol Infect Dis 75: 271–276. 23276772 10.1016/j.diagmicrobio.2012.11.021 PMC4629818

[28] van Gils EJ, Veenhoven RH, Hak E, Rodenburg GD, Keijzers WC, et al (2010) Pneumococcal conjugate vaccination and nasopharyngeal acquisition of pneumococcal serotype 19A strains. JAMA 304: 1099–1106. 20823436 10.1001/jama.2010.1290

[29] Andrews NJ, Waight PA, George RC, Slack MP, Miller E (2012) Impact and effectiveness of 23-valent pneumococcal polysaccharide vaccine against invasive pneumococcal disease in the elderly in England and Wales. Vaccine 30: 6802–6808. 23000122 10.1016/j.vaccine.2012.09.019

[30] Trück J, Lazarus R, Clutterbuck EA, Bowman J, Kibwana E, et al (2012) The zwitterionic type I Streptococcus pneumoniae polysaccharide does not induce memory B cell formation in humans. Immunobiology 218: 368–372. 22704520 10.1016/j.imbio.2012.05.008

[31] Poolman J, Kriz P, Feron C, Di-Paolo E, Henckaerts I, et al (2009) Pneumococcal serotype 3 otitis media, limited effect of polysaccharide conjugate immunisation and strain characteristics. Vaccine 27: 3213–3222. 19446194 10.1016/j.vaccine.2009.03.017

[32] Rodrigues F, Foster D, Caramelo F, Serranho P, Gonçalves G, et al (2012) Progressive changes in pneumococcal carriage in children attending daycare in Portugal after 6 years of gradual conjugate vaccine introduction show falls in most residual vaccine serotypes but no net replacement or trends in diversity. Vaccine 30: 3951–3956. 22480926 10.1016/j.vaccine.2012.03.058

