# Supplementary material for: Pneumococcal Serotype-Specific Antibodies Persist through Early Childhood after Infant Immunization: Follow-Up from a Randomized Controlled Trial
Source: PLoS One. 2014 Mar 11;9(3):e91413. doi: 10.1371/journal.pone.0091413 (PMC3950188; doi:10.1371/journal.pone.0091413)
Supplement: Protocol S1 — Trial Protocol (PDF) [file pone.0091413.s002.pdf]

Study Title: A follow-on, multi-centre, open-label, clinical, phase 4 trial to investigate the persistence of serotype-specific antibodies at 40 months of age in children who have received either the 7-valent or the 13-valent pneumococcal conjugate vaccine at 2, 4 and 12 months of age and assessing the immunogenicity of a 13-valent pneumococcal conjugate vaccine booster dose given at 40 months of age.

Internal Reference No: 2009/04

Ethics Ref: 10/H0606/9

Date and Version No:27th April 2010, version number 3.

Chief Investigator: **Professor Andrew Pollard** – Professor of Paediatric Infection and Immunity and Hon Consultant, Oxford University.

Investigators: **Professor Adam Finn** - Head of the Academic Unit of Child Health and Hon Consultant in Paediatric Infectious Diseases and Immunology, Bristol University

Dr Saul Faust - Senior Lecturer in Paediatric Infectious Diseases & Immunology, Southampton University

Dr Paul Heath - Hon Consultant in Paediatric Infectious Diseases, St George's, University of London and Vaccine Institute in London.

Dr Matthew Snape – Consultant in Vaccinology and Hon Consultant Paediatrician, Oxford University

Sponsor: University of Oxford

Funder: Pfizer (formerly Wyeth) and the NIHR Oxford Biomedical Research Centre.

Signature: 29/4/10

TABLE OF CONTENTS

1. AMENDMENT HISTORY 4
2. SYNOPSIS 4
3. ABBREVIATIONS 9
4. Background and Rationale 11
5. OBJECTIVES 14
   - 5.1 Primary Objective 14
   - 5.2 Secondary Objectives 14
6. STUDY DESIGN 16
   - 6.1 Summary of Study Design 16
   - 6.2 Primary and Secondary Endpoints/Outcome Measures 17
   - 6.3 Study Participants 18
7. STUDY PROCEDURES 20
   - 7.1 Informed Consent: 20
   - 7.2 Screening for eligibility and study discussion: 21
   - 7.3 Baseline assessments: 21
   - 7.4 Subsequent assessments: 22
   - 7.5 Visit 1: 23
   - 7.6 Visit 2 (28 to 42 Days After Visit 1) 24
8. Study Duration 25
   - 8.1 Definition of End of Study 25
   - 8.2 Discontinuation/Withdrawal of Participants from Study Treatment: 25
9. SOURCE DATA 26
10. Treatment of trial participants 27
    - 10.1 Interventions: 27
11. STORAGE OF STUDY TREATMENT 28
12. COMPLIANCE WITH STUDY TREATMENT 29
13. ACCOUNTABILITY OF STUDY TREATMENT 29
14. CONCOMITANT MEDICATION 30
15. LABORATORY ANALYSIS 30
16. SAFETY 32
    - 16.1 Adverse Event (AE) 32
    - 16.2 Adverse Reaction (AR) 32
    - 16.3 Medically significant adverse events 32
    - 16.4 Unexpected Adverse Reaction 33
    - 16.5 Serious or Severe Adverse Events 33

16.6 Serious Adverse Event or Adverse Drug Reaction 33
16.7 Expected Adverse Reactions 34
16.8 Suspected Unexpected Serious Adverse Reactions (SUSAR) 34
17. Causality Assessment 34
18. Reporting Procedures for Serious Adverse Events 35
18.1 Reporting Procedures for All Adverse Events 36
18.2 Solicited Reactions 36
18.3 General Reactions 38
18.4 Unsolicited Reactions 38
18.5 Other reportable information 39
19. STATISTICS AND ANALYSIS 40
19.1 Number of Participants 40
19.2 Description of statistical methods 40
19.3 Level of statistical significance 41
19.4 Analysis and endpoints 41
20. DIRECT ACCESS TO SOURCE DATA/DOCUMENTS 42
21. QUALITY CONTROL AND QUALITY ASSURANCE PROCEDURES 42
22. ETHICS 43
22.1 Declaration of Helsinki 43
22.2 CH Guidelines for Good Clinical Practice 43
22.3 Other Ethical Considerations 43
23. PARTICIPANT CONFIDENTIALITY 44
24. COMPENSATION FOR HARM 44
25. DATA HANDLING AND RECORD KEEPING 44
26. FINANCING AND INSURANCE 45
27. APPENDIX A: STUDY FLOW CHART 46
28. REFERENCES 46

1. AMENDMENT HISTORY

Amendment No.	Protocol Version No.	Date issued	Author(s) of changes	Details of Changes made
1	2	6th April 2010	Dr Matthew Snape	See below
2	3	27th April	Dr Matthew Snape	See below

Amendment 1

- Study timelines amended to study starting in April, rather than March 2010
- Background to the study amended to reflect recent incorporation of the 13-valent pneumococcal conjugate vaccine into the routine infant immunisation schedule in the UK
- incorporation of the possibility that sites other than Oxford will be able to provide samples for B cell analysis, by the use of frozen blood samples
- changes to the methods of informing parents of their child's results from the previous study and inviting them to participate in this new study; this has now been changed to contacting all families by mail with a follow-up telephone call shortly after.
- the refinement of temporary exclusion criteria relating to receipt of recent immunisations, to distinguish the exclusion period for live vaccines (remains at 28 days) and non-live vaccines (reduced to 7 days).
- change to the needle to be used for vaccine administration to 0.6 x 25 mm 23 gauge needle; the previous needle size of 21 gauge was written in error.
- change to the handling of blood samples from participants whose B cell response to immunisation is to be assessed, such that for these participants any blood sample up to 5ml will be collected into 'red top' serum separator tubes and any sample beyond 5ml will be stored in a heparinised tube for B cell analysis.

- the definition of a lack of response to he PCV13 vaccine received in this study has been formalised to state that this refers to a PCV13 serotype specific IgG < 0.35 mcg/ml

Amendment 2

- As PCV13 is now a licensed vaccine in the UK, it is felt that a safety review by a study specific Data Monitoring Committee was no longer required for this study. Instead review of any SAEs will be performed by the Oxford Radcliffe Hospitals Trust / University of Oxford Trial Safety Committee (TSG). This change has been reflected in sections 18 and 21.

- The B cell section of laboratory methods (section 15) has been altered to allow the analysis of B cell responses to vaccines received in the study to be preformed at local study sites as well as at Oxford.

2. SYNOPSIS

**Study Title**	**A follow-on, multi-centre, open-label, clinical, phase 4 trial to investigate the persistence of serotype-specific antibodies at 40 months of age in children who have received either the 7-valent or the 13-valent pneumococcal conjugate vaccine at 2, 4 and 12 months of age and assessing the immunogenicity of a 13-valent pneumococcal conjugate vaccine booster dose given at 40 months of age.**
**Internal ref. no.**	2009/04
**Study Design**	Follow-on study from a randomised controlled study
**Study Participants**	Participants who have completed the Wyeth-sponsored PCV13 infant trial study (6096A1-007)
**Number of Participants**	Approximately 100
**Planned Study Period**	April 2010 to April 2011
**Primary Objective**	To assess the proportion of participants, immunised with the 13-valent pneumococcal conjugate vaccine (PCV13) at 2, 4 and 12

	months of age, who have IgG concentrations ≥ 0.35mcg/ml for PCV13 serotypes at the time when preschool booster vaccinations are due (at 40 months* of age).
**Secondary Objectives**	To assess the proportion of participants, immunised with the 7-valent pneumococcal conjugate vaccine (PCV7) at 2, 4 and 12 months of age, who have IgG concentrations ≥ 0.35mcg/ml for PCV13 serotypes at the time when preschool booster vaccinations are due (at 40 months* of age) and comparing these to the proportion of participants achieving this threshold after infant immunisation with PCV 13.<br><br>To assess and compare PCV13 serotype-specific IgG geometric mean concentrations (GMCs), opsonophagocytic activity (OPA) geometric mean titres (GMTs) and the proportion of participants with PCV13 serotype-specific OPA titres ≥ 1:8 at 40 months* of age in children immunised in infancy with either PCV7 or PCV13.<br><br>To assess and compare PCV serotype-specific IgG GMCs, OPA GMTs and the proportion of participants with IgG concentrations ≥ 0.35mcg/ml and OPA titres ≥ 1:8 one month following a booster dose of PCV13 at 40 months* of age in children previously immunised with PCV7 and PCV13 at 2, 4 and 12 months of age.<br><br>To determine reactogenicity of the pre-school PCV13 booster in terms of rates of local and systemic reactions following vaccination.<br><br>To investigate the influence of genetic polymorphisms on the above immunological markers following infant immunisation with PCV7 or PCV13 and following a booster dose of PCV13 at 40 months* of age and on the nature of adverse reactions observed after this booster immunisation.

	To measure the pneumococcal serotype-specific memory B cells frequencies before and 1 month after a dose of PCV13 at 40 months* of age in a subset of children previously immunised with PCV7 or PCV13 at 2, 4 and 12 months of age (serotype studies to include serotypes including 4, 14, 23F (present in PCV7) and, 1, 3, 19A).
**Primary Endpoint**	The proportion of participants with PCV13 serotype-specific IgG concentrations ≥ 0.35mcg/ml at 40 months* of age following immunisation with PCV13 at 2, 4 and 12 months of age.
**Secondary Endpoints**	The proportion of participants with PCV13 serotype-specific IgG concentrations ≥ 0.35mcg/ml at 40 months* of age following immunisation with PCV7 at 2, 4 and 12 months of age.<br><br>The PCV13 serotype-specific IgG GMCs, OPA GMTs and proportion of participants with OPA titres ≥ 1:8 at 40 months* of age in children primed with either PCV7 or PCV13.<br><br>The PCV13 serotype-specific IgG GMCs, OPA GMTs and proportion of participants with IgG concentrations ≥ 0.35mcg/ and OPA titres ≥ 1:8 1 month following a dose of PCV13 at 40 months* of age in children who have received PCV7 or PCV13 at 2, 4 and 12 months of age.<br><br>Rates of local and systemic reactions (reactogenicity) following vaccination with the pre-school PCV13 booster at 40 months*.<br><br>The identification of genetic polymorphisms in influencing the above immunological markers and adverse reactions to vaccines.<br><br>The frequency of PCV13 specific memory B cells before and after immunisation at 40 months* of age determined by B cell ELISPOT and, where sufficient B cells are available for analysis,

	phenotyping of these cells.
**Intervention (s)**	Immunisation with the 13-valent pneumococcal conjugate vaccine (PCV13) at approximately 40 months* of age.

* ages are approximate

3. ABBREVIATIONS

AE	Adverse Event
ADR	Adverse Drug Reaction
AR	Adverse Reaction
CI	Chief Investigator
CRF	Case Report Form
CTRG	Clinical Trials & Research Governance, University of Oxford
GCP	Good Clinical Practice
GMC	Geometric Mean Concentration
GMT	Geometric Mean Titres
ICF	Informed Consent Form
ICH	International Conference on Harmonisation
IMP	Investigational Medicinal Product
IgG	Immunoglobulin G
MenC	Serogroup C meningococcus
NIHR	National Institute of Health Research
NRES	National Research Ethics Service
OPA	Opsonophagocytic Assay
PCV7	7-valent Pneumococcal Vaccine
PCV13	13-valent Pneumococcal Vaccine
PI	Principal Investigator
PIL	Participant/ Patient Information Leaflet
R&D	NHS Trust R&D Department
REC	Research Ethics Committee

SAE	Serious Adverse Event
SAR	Serious Adverse Reaction
SBA	Serum bactericidal antibodies
SOP	Standard Operating Procedure
SUSAR	Suspected Unexpected Serious Adverse Reactions

4. BACKGROUND AND RATIONALE

Infections caused by ***Streptococcus pneumoniae*** are a major cause of morbidity and mortality in infants and children worldwide. WHO estimates that globally, pneumococcus accounts for over 1 million childhood (under five years) deaths per year[1].

Bacteraemic pneumonia is the most common form of invasive pneumococcal disease (IPD) and is the most common cause of pneumococcal death worldwide[2]. The next most common cause of death from pneumococcal disease is pneumococcal meningitis, with or without bacteraemia. Pneumococcal meningitis is a severe disease with high mortality and high incidence of neurological sequelae[3]. Non-invasive pneumococcal disease including otitis media, sinusitis, bronchitis, and non-bacteraemic pneumonia are much more common than invasive disease, but are much less severe.

Most children become colonised with ***Streptococcus pneumoniae*** within the first two years of life. Asymptomatic nasopharyngeal carriage is significant as it plays a part in both the spread of the organism, as well as the development of invasive disease. In industrialised countries, virtually every child becomes a nasopharyngeal carrier of S. pneumoniae during the first year of life[4].

Before the introduction of routine childhood immunisation against pneumococcal disease in 2000, the incidence of IPD in the United States was approximately 96/100,000 cases in children less than 5 years of age, with higher rates in children less than 2 years of age[5]. Reported IPD rates in Europe have generally been lower, ranging from 11/100,000 in Germany to 56/100,000 in those less than 5 years of age in Spain, with higher rates in children under 2 years of age[6]. Differences between regions in the reported incidence of invasive disease may be due to different laboratory practices in culturing blood.-This is supported by the similar rates of pneumococcal meningitis in the United States and Europe, a disease for which there is little difference in regional methods.

A 23-valent polysaccharide (PS) vaccine has been available for many years, but as with other PS vaccines, it is not effective in infants. PS antigens are T-cell independent and poorly immunogenic in the immature immune systems of infants[7].

Conjugation of the pneumococcal PS to a protein converts the PS to a T-cell dependent antigen, allowing infants to mount an effective immune response. Using this technology, a 7-valent pneumococcal conjugate vaccine (PCV7) was developed by Wyeth vaccines (now Pfizer). PCV7 contains PS from serotypes 4, 6B, 9V, 14, 18C, 19F and 23F, the pneumococcal serotypes responsible for the majority of IPD in industrialised countries, conjugated to non-toxic diphtheria toxin cross-reacting material (CRM_{197}). Controlled clinical trials with PCV7 show that it is effective in preventing IPD and pneumonia due to serotypes in the vaccine[8].

Since licensure of PCV7 in the United States in 2000, its effectiveness in preventing IPD in infants has been definitively documented[9]. Data from the Active Bacterial Core Surveillance (ABC) network show that in 2001, the incidence of IPD in children less than 5 years of age had declined by 56%, and in 2004 by 76%, with a 96% decline in IPD caused by vaccine serotypes. In addition to this direct effect of the vaccine, vaccination of children has also reduced the incidence of disease in adults[10]. There were statistically significant declines in IPD in adults 20 years and older after the introduction of PCV7, the largest in those greater than 65 years. The absolute number of cases prevented by this indirect effect of PCV7 is actually estimated to be larger than the direct effect, which makes universal vaccination highly cost effective[10, 11].

The ABC data have also shown small, but statistically significant increases in pneumococcal disease due to non-vaccine serotypes in both infants and adults. Serotypes 19A, 3, 15, 22F, 33F, and 35 have all been isolated, with 19A being the most prevalent. Genetic typing of serotype 19A strains identified the presence of the primary clone seen before the introduction of PCV7, as well as new genetic types previously associated with other, principally vaccine serotypes; this appears to be the result of capsular switching[12.]

Before introduction of PCV7, vaccine serotypes were responsible for approximately 80% to 90% of IPD in the USA, 60% to 80% of IPD in Europe, and 40% to 80% of IPD in other regions of the world. To expand the percentage of IPD that could be prevented, a 13-valent pneumococcal conjugate vaccine (PCV13) containing the 7 serotypes in Prevenar and 6 additional pneumococcal conjugate serotypes (1, 3, 5, 6A, 7F, and 19A) was licensed for use in the EU in December 2009[13].

The Health Protection Agency (HPA) surveillance data suggests that the cases of IPD in children under 5 years of age in England and Wales dropped by 41% from the time of introduction of PCV7 in 2006 to the end of the epidemiological year 2007/2008[14]. Analysis of this data suggests that the 13-valent vaccine would cover approximately 74% of current IPD in children under 5 years of age and approximately 67% of the remaining disease in people ≥ 5 years of age. IPD caused by PCV7-covered serotypes is very low in children under 5 years of age and, of the IPD remaining in England and Wales, an extra 50% is potentially preventable in children by switching to PCV13. Interestingly, the incidence of disease due to non-vaccine serotypes has been increasing in the last few years and the serotype replacement remains a concern, especially serotypes 7F, 19A and 22F.

Several UK sites have recently taken part in a parallel-group, randomized, active-controlled, double-blind, multicenter trial to evaluate the safety, tolerability, and immunogenicity of a 13-valent pneumococcal conjugate vaccine in healthy infants given with routine paediatric vaccinations. In the comparison arm of this study, infants received PCV7. Data from this study suggest PCV13 was immunogenic and well tolerated when given as part of the UK infant vaccine course. Further, the percentages of participants achieving the thresholds of response for the concomitant antigens were comparable between the two groups. For the 6 additional serotypes not contained in PCV7, PCV13 vaccination at 2, 4 and 12 months resulted in 88.2% - 100% of vaccines achieving the correlate of protection[15].

The duration of serotype-specific antibody persistence following primary immunisation with PCV13 is not known. Further, there are no data on the serotype-specific IgG persistence to 40 months following a 2, 4 and 12 month schedule with PCV7. Although the largest burden of pneumococcal disease is in the under 2 year olds, there is also a significant burden of invasive pneumococcal disease in the 2 to 5 year old age group. This age group is also important for the carriage and spread of pneumococcus; hence, pneumococcal serotype-specific immunity of this age-group is likely to be important in reducing disease burden and enhancing herd immunity. It would therefore be important to ensure that serotype-specific antibodies persist following a primary vaccination program, and if not, to assess the immune response to a pre-school booster of pneumococcal vaccine.

PCV13 has recently replaced PCV7 in the UK immunisation schedule and an ongoing enhanced surveillance program will monitor the impact of this change on the incidence of the additional 6 serotypes contained in PCV13. One option to further enhance the control of these serotypes is the administration of a pre-school booster dose of PCV13 to those children immunised in infancy with PCV7, however, at present there are no immunogenicity data to inform such an option.

An important functional measure of protective immunity induced by pneumococcal vaccines appears to be the assay for opsonic capacity, which is the host's primary mechanism of protecting itself against pneumococcal infections; pneumococcal vaccines are designed to induce opsonic antibodies[16]. We will therefore use a standardised opsonophagocytosis assay OPA methodology to evaluate the protective immunity induced by the PCV7 and PCV13 schedules employed in these studies in addition to measuring serotype specific IgG concentrations.

We have previously found that the B cell response to conjugate vaccines generates memory cells which, in infants, predict both the persistence of antibody and the response to booster doses of vaccines[17]. There is some variation in the responses to different serotypes in PCV13 and we have developed B cell assays that can be used to explore the quantity and phenotype of these responses[18]. We will use these assays to describe the B cell response to a booster dose of PCV13.

5. OBJECTIVES

5.1 Primary Objective

To assess the proportion of participants, immunised with the 13-valent pneumococcal conjugate vaccine (PCV13) at 2, 4 and 12 months of age, who have IgG concentrations ≥ 0.35mcg/ml for PCV13 serotypes at the time when preschool booster vaccinations are due (at 40 months* of age).

5.2 Secondary Objectives

To assess the proportion of participants, immunised with the 7-valent pneumococcal conjugate vaccine (PCV7) at 2, 4 and 12 months of age, who have IgG concentrations ≥ 0.35mcg/ml for PCV13 serotypes at the time when preschool booster vaccinations are due (at 40 months* of age) and comparing these to the

proportion of participants achieving this threshold after infant immunisation with PCV 13.

To assess and compare PCV13 serotype-specific IgG geometric mean concentrations (GMCs), opsonophagocytic activity (OPA) geometric mean titres (GMTs) and the proportion of participants with PCV13 serotype-specific OPA titres ≥ 1:8 at 40 months* of age in children immunised in infancy with either PCV7 or PCV13.

To assess and compare PCV serotype-specific IgG GMCs, OPA GMTs and the proportion of participants with IgG concentrations ≥ 0.35mcg/ml and OPA titres ≥ 1:8 one month following a booster dose of PCV13 at 40 months* of age in children previously immunised with PCV7 and PCV13 at 2, 4 and 12 months of age.

To determine reactogenicity of the pre-school PCV13 booster in terms of rates of local and systemic reactions following vaccination.

To investigate the influence of genetic polymorphisms on the above immunological markers following infant immunisation with PCV7 or PCV13 and following a booster dose of PCV13 at 40 months* of age and on the nature of adverse reactions observed after this booster immunisation.

To measure the pneumococcal serotype-specific memory B cells frequencies before and 1 month after a dose of PCV13 at 40 months* of age in a subset of children previously immunised with PCV7 or PCV13 at 2, 4 and 12 months of age (serotype studies to include serotypes including 4, 14, 23F (present in PCV7) and, 1, 3, 19A).

* Ages are approximate

6. STUDY DESIGN

6.1 Summary of Study Design

This is a follow-on, multi-centre, open-label, clinical trial. The purpose of this trial is to investigate the concentrations of serotype-specific antibodies to IgG included in PCV13 in children who have received either the PCV7 or PCV13 primary immunisation at 2, 4 and 12 months of age. We intend to recruit all interested participants who completed the Wyeth-sponsored PCV13 infant trial study (6096A1-007) at selected study sites (i.e. those that recruited the majority of the children in the original study). The study will start in April 2010, at which time the eldest participants in the 6096A1-007 study will be approximately 43 months of age. There will be two visits per participant, 1 month apart from each other. At visit one, all participants will have a blood test and receive a dose of PCV13. At visit 2, all participants will have a blood test and will be offered the remaining pre-school booster vaccinations unless they have already received them. As initial enrolment into the first study was over 8 months, V1 for this follow-up study is scheduled to finish in October 2010. The scheduled visits of all participants recruited for this study should be completed by December 2010, however the end of study will be considered the end of processing samples for laboratory testing, which is likely to continue until April 2011.

With specific consent, a blood clot from the serum samples will be used to store DNA for analysis of the genetic associations with immune responses and adverse reactions to vaccines.

Available whole blood from children at study sites including Oxford and Southampton will be used for B cell assays.

Of the 286 children randomised in the original study, approximately 240 were enrolled in the sites that will be participating in this follow on study. Of these, approximately 200 completed the study. It is anticipated that 50% of these participants are likely to take part in this follow-on study, therefore the study population is likely to be approximately 100 children.

The results from the 13 month blood test from the original study have clinical relevance and will be relayed to the parent/ guardian of any child that participated in the original study in a letter that will also give details of this follow-on study and invite them to take part. In order to help parents interpret these results, and to answer any questions they may have about this follow –on study, we will telephone the families of all participants in the weeks following the sending of these letters unless they have requested that we do not do so.

6.2 Primary and Secondary Endpoints/Outcome Measures

Primary Endpoint

The proportion of participants with pneumococcal serotype-specific IgG concentrations ≥ 0.35mcg/ml at 40 months* of age following immunisation with PCV13 at 2, 4 and 12 months of age.

Secondary Endpoint

The proportion of participants with PCV13 serotype-specific IgG concentrations ≥ 0.35mcg/ml at 40 months* of age following immunisation with PCV7 at 2, 4 and 12 months of age.

The PCV13 serotype-specific IgG GMCs, OPA GMTs and proportion of participants with OPA titres ≥ at 40 months* of age in children primed with either PCV7 or PCV13.

The PCV13 serotype-specific IgG GMCs, OPA GMTs and proportion of participants with IgG concentrations ≥ 0.35mcg/ml and OPA titres ≥ 1:8, 1 month following a dose of PCV13 at 40 months* of age in children who have received PCV7 or PCV13 at 2, 4 and 12 months of age.

Rates of local and systemic reactions (reactogenicity) following vaccination with the pre-school PCV13 booster at 40 months*.

The identification of genetic polymorphisms influencing the above immunological markers and adverse reactions to vaccines.

The frequency of PCV13 specific memory B cells in a subset of participants before and after immunisation at 40 months* of age as determined by B cell ELISPOT and where sufficient B cells are available for analysis, phenotyping of these cells.
* Ages are approximate.

6.3 Study Participants

6.3.1 Overall Description of Study Participants

Healthy participants who completed the Wyeth-sponsored PCV13 infant trial study (6096A1-007) at the study sites participating in this follow on study who are currently aged 39-46 months.

6.3.2 Inclusion Criteria

Participants must meet the following conditions in order to be enrolled:

1. Participant completed the Wyeth-sponsored PCV13 infant trial study (6096A1-007) at one of the study sites participating in this follow-on study.
2. Aged 39-46 months (inclusive) at time of enrolment.
3. Available for entire study period and whose parent/legal guardian can be reached by telephone.
4. Healthy children as determined by medical history, physical examination, done by a study nurse (and/or study doctor if required, depending on the medical history of the participant and physical assessment), and judgment of the investigator.
5. Parent/legal guardian must be able to complete all relevant study procedures during study participation.

6.3.3 Exclusion Criteria

Participants with any of the following conditions or characteristics will be excluded from study enrolment:

1. Has received further doses of pneumococcal vaccination with licensed or investigational pneumococcal vaccine <u>other than</u> those given as part of the Wyeth-sponsored PCV13 infant trial study (6096A1-007).

2. A previous anaphylactic reaction to any vaccine or vaccine-related component.
3. Contraindication to vaccination with pneumococcal conjugate vaccine.
4. Bleeding diathesis or condition associated with prolonged bleeding time that would contraindicate intramuscular injection.
5. Known or suspected immune deficiency or suppression.
6. History of culture-proven invasive disease caused by S pneumoniae.
7. Major known congenital malformation or serious chronic disorder.
8. Significant neurologic disorder or history of seizures including febrile seizure, or significant stable or evolving disorders such as cerebral palsy, encephalopathy, hydrocephalus, or other significant disorder.
9. Receipt of blood products or gamma-globulin (including hepatitis B immunoglobulin and monoclonal antibodies; eg, synagisB).
10. Participation in another investigational study other than the Wyeth-sponsored PCV13 infant trial study (6096A1-007). Participation in purely observational studies is acceptable.
11. Child who is a direct descendant (child, grandchild) of the study site personnel.

6.3.4 Temporary exclusion criteria

1. In the event of systemic illness or fever > 38 degrees centigrade at the time of the visit, immunisation will be deferred and rearranged as appropriate when the participant is recovered.

2. Any live immunisation within 28 days prior to enrolment, or any other (non-live) vaccine within the 7 days prior to enrolment.

6.3.5 Expenses and Benefits

1. All the study visits will be conducted at the participant's home or most convenient place to the participant's parent/legal guardian.

2. None of the participants will receive economical reimbursement for their participation in the study.

3. Participants will have the benefit of receiving a Pneumococcal vaccine protecting against 13 serotypes and having their immune response to the vaccine investigated by serology tests.

7. STUDY PROCEDURES

7.1 Informed Consent:

Informed consent will be taken by members of the research team, either a doctor or nurse trained in taking informed consent. The parent/guardian must personally sign the latest approved version of the informed consent form before any study-specific procedures are performed.

Written and verbal versions of the participant information and informed consent will be presented to the participant's parent/guardian detailing no less than: the exact nature of the study; the implications and constraints of the protocol; the known side effects and any risks involved in taking part. It will be clearly stated that the participant is free to withdraw from the study at any time for any reason without prejudice to future care, and with no obligation to give the reason for withdrawal.

The parent/guardian will be allowed as much time as wished to consider the information, and the opportunity to question the Investigator, or other independent parties to decide whether they will participate in the study. Written Informed Consent will then be obtained by means of parent/guardian dated signature and dated signature of the person who presented and obtained the informed consent. A copy of the signed Informed Consent will be given to the parent/guardian. The original signed form will be retained at the study site.

In the event that the parent/guardian is illiterate then a third party may act as an impartial witness for the parent/guardian to attest that the information in the consent form and any other written information was accurately explained to, and apparently understood by, the parent/guardian and that informed consent was freely given by

the parent/guardian. In this event the impartial witness will also sign and date the consent form.

7.2 Screening for eligibility and study discussion:

Parent’s who had not previously expressed an unwillingness to be contacted about this follow - on study will be sent a letter providing their child’s blood test results from the initial study and to inform them of this follow-on study. In order to help parents interpret these results, and to answer any questions they may have about this follow –on study, we will telephone the families of all participants in the weeks following the sending of these letters unless they have requested that we do not do so. If parents show an interest in enrolling their child in this study we will ask screening questions to assess their child’s eligibility to take part in this follow-on study. If these screening questions do not identify any ineligibility to take part in the study, and the parent wishes their child to participate, an appointment for V1 will be arranged.

If the blood test from 13 months in the original study demonstrated that a child had MenC SBA titres < 1:8, anti PRP (Hib) IgG < 0.15 mcg/ml or PCV7 pneumococcal serotype specific IgG < 0.35 mcg/ml and they are not taking part in this study then an offer will be made to immunise the child with the relevant vaccine through the clinical teams at the relevant study sites (outside this study protocol). If the child does take part in this study and they have MenC SBA titres < 1:8 or anti PRP (Hib) IgG < 0.15 mcg/ml then the study team will offer to immunise the child with the relevant vaccine at visit 2 of this study (or, if preferred a separate visit 3). The response to this vaccine does not form part of the study evaluation.

7.3 Baseline assessments:

7.3.1 Demographics

The date of birth, gender, ethnicity and address will be recorded.

7.3.2 Medical History

Information regarding medical history, previous hospital admissions, surgical interventions will be recorded.

7.3.3 Concomitant Medication

All current prescription medication and any vaccines received after conclusion of 6096A1-007 will be recorded.

7.3.4 Physical Examination

1. A brief physical assessment of the child will be carried out on visit 1 by a study nurse or doctor. If specific concerns are raised by this assessment or the child's medical history, then a formal physical examination will be performed by a study doctor.

2. Axillary temperature will be checked and recorded; this should be <38 degrees centigrades before any vaccine is administered.

3. Eligibility criteria as outlined in the inclusion/ exclusion criteria above will be applied, and consent obtained prior to recruitment into the study.

7.4 Subsequent assessments:

1. Inclusion/Exclusion criteria will be checked on each subsequent visit following Visit 1.

2. Axillary temperature will be checked on every visit before any vaccine is given.

3. The source document will be updated on each visit, recording any medical advice sought, any adverse event and concomitant medications started since the last visit.

7.4.1 Study Schedule:

We anticipate the study population will be of approximately 100 participants within all study sites.

Visit 1 will take place between April and October 2010. Visit 2 will take place approximately 4 weeks after Visit 1 between May and December 2010.

7.5 Visit 1:

1. 39-46 months inclusive.
2. Study explanation provided.
3. Obtain written informed consent from parent/legal guardian
4. Obtain and record medical and vaccination history
5. Check inclusion and exclusion criteria.
6. Perform physical examination and record findings.
7. Measure and record the participant's axillary temperature.
8. If participant is suitable for inclusion in study, assign participant number
9. Collect a blood sample (up to 10 mL) after application of topical anaesthetic cream (Ametop or, if sensitive to Ametop, EMLA)
10. Administer a single 0.5-mL intramuscular injection of PCV13 into the left deltoid (or non-dominant arm if left handed).
11. Observe the participant for at least 15 minutes after vaccination for any significant acute reactions. Any AEs noted during the observation period should be recorded on the source documents and on the AE section of the case report form (CRF).
12. Issue a ruler and a digital thermometer to the parent/legal guardian and provide instructions on their use.
13. Issue a participant diary to the parent/legal guardian and provide instruction on its completion.
14. Record vaccination details in the participant's red book.

15. Ask the parent/legal guardian to contact the investigator immediately if any significant illness or hospitalization occurs during the study period, or if the participant experiences a large (> 14 millimeters) local reaction.
16. Provision of topical anaesthetic cream for parent/ guardian to apply prior to next visit (written instructions and appropriate dressings to be provided)
17. Schedule visit 2 for 28-42 days after visit 1.
18. The investigator or an authorised designee completes the CRF and updates the vaccine accountability records.

7.6 Visit 2 (28 to 42 Days After Visit 1)

1. Review the participant's diary data since the previous visit.
2. Based on review of the participant's diary and clinical evaluation, determine whether any unsolicited AEs or serious adverse events (SAEs) have occurred since the last study visit and record them on the CRF.
3. Measure and record the participant's axillary temperature.
4. Ensure the participant continues to meet the criteria for continued participation in the trial.
5. Collect a blood sample (approximately 10 mL).
6. Administer a single intramuscular dose of Repevax or Infanrix-IPV and the MMR vaccine into the deltoid of the left and right upper arm respectively if not already received as part of standard of care.
7. Observe the participant for at least 15 minutes after vaccination for any significant acute reactions. Any AE noted during the observation period should be recorded on the source documents and on the AE section of the CRF.
8. The investigator or an authorised designee completes the CRF and updates the test article accountability records.

The study procedures are summarized in the table below:

	40 months*	41 months*
Study visits	V1<br>Recruitment	V2
Immunisations	PCV13	DtaP/IPV<br>MMR
Blood samples	√	√

* Ages are approximate

N.B. Participants in this follow-on study known to have MenC SBA titres < 1:8 at the 13 month blood sample from the original study will be offered an additional dose of MenC vaccine as part of this study. This vaccine is not to be administered concomitantly with PCV13, but can be administered at visit 2 or, if preferred by parents, an additional study visit to administer this vaccine can be performed. This vaccine will not form part of the study assessments.

8. STUDY DURATION

The start date of this study will be April 2010, anticipating its end date in April 2011.

8.1 Definition of End of Study

The study will be considered ended when all the study visits have been completed and all the biological samples processed to be read for analysis.

8.2 Discontinuation/Withdrawal of Participants from Study Treatment:

1. The parent/legal guardian of the participating children have the right to withdraw their child from the study at any time, without having to provide any particular reason for doing so.

2. Some children may be withdrawn from the study for the following reasons:

   a) Consent withdrawn.
   b) Lost to follow up.

3. If a participant is required to withdraw from the study either due to an AE during the study or an AE present at the end of it, appropriate follow up will be provided until its satisfactory resolution or stabilisation.

4. The reason for withdrawal will be recorded in the source document/CRF.

   The investigator should aim to retain as many participants within the study as possible to respect the intention to treat analysis, but may consider excluding participants who meet the following criteria from the per-protocol analysis:

   a) Inegibility (either arising during the study or retrospectively having been overlooked during screening.
   b) Significant protocol deviation.
   c) Significant non-compliance to treatment regimen or study requirements.
   d) An adverse event which requires discontinuation of the study treatment or results in inability to comply with study procedures.
   e) Disease diagnosis or progression requiring discontinuation of the study treatment or results in inability to comply with study procedures.

9. SOURCE DATA

Source documents are original documents, data, and records from which the participant's CRF data are obtained. These include, but are not limited to hospital records, (from which medical history and previous and concurrent medication may be summarised into the CRF), clinical and office charts, laboratory records and correspondence.

CRF entries will be considered source data if the CRF is the site of the original recording (e.g., there is no other written or electronic record of data). In this study the CRF will be used as the source document for assessments and measurements made at study visits. All documents will be stored safely in confidential conditions. On all study-specific documents, other than the initial response form from the participant and the signed consent, the participant will be referred to by the study participant number, not by name.

10. TREATMENT OF TRIAL PARTICIPANTS

10.1 Interventions:

Study vaccine

10.1.1 13-valent Pneumococcal Conjugate Vaccine

PCV13 contains saccharides from pneumococcal serotypes 1, 3, 4, 5, 6A, 6B, 7F, 9V, 14, 18C, 19A, 19F, and 23F individually conjugated to cross-reacting material (CRM_{l97}). The vaccine is formulated to contain 2.2 pg of each saccharide, except for 4.4 pg of 6B, with a total nominal dose of 29 pg CRM197 per 0.5-mL dose. The final formulation contains 5 mM succinate buffer, with 0.125 mg of aluminum as aluminum phosphate per 0.5-mL dose. The vaccine is administered intramuscularly by injecting 0.5 mL into the non dominant deltoid muscle at Visit 1.

Vaccines administered concomitantly as part of standard of care

10.1.2 Repevax

1 dose (0.5 mL) of Repevax contains; Diphtheria toxoid ≥2IU (2 Lf); Tetanus toxoid ≥ 20 IU (5 Lf); Pertussis antigens; Pertussis toxoid - 2.5 mcg; Filamentous haemagglutinin 5mcg, Fimbriae types 2 + 3 5 mcg, Pertactin 3mcg, Poliomyelitis virus type 1** (inactivated) 40 D antigen units; Poliomyelitis virus type 2(inactivated) 8 D antigen units; Poliomyelitis virus type 3** (inactivated); 32 D antigen units;

Adsorbed on aluminium phosphate1.5 mg (0.33 mg Al). The vaccine is administered intramuscularly by injecting 0.5 mL into the deltoid muscle at visit 2.

If Repevax is not available for supply for routine immunisation, then an alternative vaccine for pre-school immunisation, Infanrix-IPV, will be used.

10.1.3 MMR

Each 0.5 ml dose when reconstituted contains not less than equivalent of: 1,000 TCID50 *of Measles Virus Live (the more attenuated Enders Line of the Edmonston strain). 20,000 TCID50 of Mumps Virus Live (Jeryl Lynn® Level B strain). 1,000 TCID50 of Rubella Virus Live (Wistar, RA 27/3 Strain). *Tissue Culture Infectious Dose. The vaccine is administered intramuscularly by injecting 0.5 mL into the opposite deltoid muscle to the combination diphtheria, tetanus, polio and pertussis vaccine at visit 2.

<u>Vaccine administered to boost immunity in response to low antibody titres from the original study</u>

10.1.4 Monovalent MenC Vaccine

One of the three monovalent MenC vaccines licensed for use in the UK will be used to boost immunity against MenC disease for this children participating in this follow-on study who had MenSBA titres < 1:8 in the original PCV13 study.

All vaccines will be administered using a 0.6 x 25 mm 23 gauge needle.

11. STORAGE OF STUDY TREATMENT

Pfizer's PCV13 vaccine will be stored at the study site following the manufacturer's recommendations.

All study vaccines will be shipped at +2 centigrades to +8 centigrades to the study site. Upon receipt at the study site, vaccines should be immediately transferred to +2 to +8 centigrades temperature-monitored refrigerator for storage.

The refrigerator must be secure and have limited access. It is the Investigator’s (or designee) responsibility to record daily refrigerator temperature readings, to maintain a temperature log for the refrigerator, and to alert the sponsor of any deviations. In case of temperature deviations, the study vaccines cannot be used and should be quarantined until authorization to use the vaccine is received from Pfizer. Guidance in temperature monitoring and procedures for review or temperature deviations will be provided.

12. COMPLIANCE WITH STUDY TREATMENT

1. A diary card will be given to each participant's parent/legal guardian on Visit 1, which should be filled in with all information required after each visit.
2. The diary will be reviewed at each visit and all relevant and necessary information will be copied into the source document/CRF.
3. The parent/legal guardian will be encouraged to comply with the eligibility criteria throughout the duration of the study as appropriate.

13. ACCOUNTABILITY OF STUDY TREATMENT

1. All doses of PCV13 vaccine will be supplied by Pfizer to the study sites.
2. A member of the Investigator’s Team will collect the study treatment.
3. All movements between Pfizer and the study site will be documented.
4. The Investigator will be responsible of ordering a new supply of the vaccine when necessary.
5. Unused/expired/damaged vaccines/replacement vials will be retrieved by the manufacturer at its agreed time/end of the study.

6. The vaccines administered concomitantly as part of standard of care or to boost immunity in response to previous low antibody levels will be supplied directly to each study site by NHS distribution routes. As these are not forming part of the study evaluation these vaccines will not require additional labeling,
7. A vaccine accountability log will be used in order to check that supplies, used and remaining vaccine numbers match at all times.
8. A temperature log will be kept up to date.
9. Cool boxes with attached thermometer will be used while transporting the study treatment during scheduled visits.
10. Storage and transport temperature will follow the manufacturer's recommendations at all times

14. CONCOMITANT MEDICATION

1. Participants should avoid taking any drugs/medications for the duration of the study, unless required for the participant's health.
2. All medications will be recorded on the source document/CRF together with the reason for starting it, the intensity, start date and stop date if applicable.
3. It is the investigator's responsibility to review the on-going eligibility of the participant during the duration of the study.

15. LABORATORY ANALYSIS

Blood samples of up to 10 mls will be collected at both study visits. If the sample collected is not to be used for B cell analysis all blood will be collected into ''red top' serum separator tubes. If the sample is to be used for B cell analysis any blood sample up to 5ml will be collected into 'red top' serum separator tubes and any sample beyond 5ml will be stored in a heparinised tube for B cell analysis.

Blood samples in the serum separator tubes will be stored at room temperature for up to 60 minutes, and then stored at between 2 to 8°C. Samples collected at each study site will be centrifuged at 3000 rpm for 10 minutes within 24 hours at the study

site and separated into at least 2 aliquots for storage at or below -30°C. Pfizer will coordinate the shipping of the aliquots required for serological analysis from study sites to their laboratories in Pearly River, USA.

1. **Antibody concentrations and Opsonophagocytic activity**

Antibody concentrations (ELISA or Luminex) and opsonophagocytic activity will be analysed by Pfizer in Pearl River, USA to provide consistency with the original study assays.

Serum concentrations of anticapsular immunoglobulin G (IgG) for all PCV13 serotypes will be identified by ELISA and expressed as micrograms per milliliter. The assay will employ 2 absorbents: a C polysaccharide-containing cell wall extract plus serotype 22Fcapsular polysaccharide. Serum concentrations of anti-capsular immunoglobulin G (IgG) for further vaccine serotypes may also be evaluated.

Should any of the participants have a PCV13 serotype-specific IgG concentration < 0.35 mcg/ml in the serum obtained at visit 2 his/her parent/legal guardian will be notified and a booster dose of PCV13 will be offered to the participant.

2. **DNA storage**

DNA will be extracted from blood clots and stored in the biobank of the Oxford Vaccine Centre laboratory at the Centre of Clinical Vaccinology and Tropical Medicine, University of Oxford or at local study sites for later analysis for genetic polymorphisms.

3. **B cell responses**

B cell responses will be determined in participants enrolled at study sites including Oxford and Southampton. At Oxford this analysis will be conducted on fresh samples. For samples obtained elsewhere the analysis will be conducted at Oxford on frozen samples transferred from the other study sites. Where applicable, the serotype-specific antigen specific B cell response to serotypes including 4, 14, 23F

(present in PCV7) and, 1, 3, 19A (present in PCV13) will be analysed in the Oxford Vaccine Centre laboratory at the Centre for Clinical Vaccinology and Tropical Medicine, University of Oxford by ELISPOT and flow cytometric assays depending on the available volumes of blood.

B cell response data will be summarized descriptively as this procedure will be observational only.

16. SAFETY

16.1 Adverse Event (AE)

An AE or adverse experience is:

Any untoward medical occurrence in a patient or clinical investigation participant administered a medicinal product, which does not necessarily have to have a causal relationship with this treatment (the study medication).
An AE can therefore be any unfavourable and unintended sign (including an abnormal laboratory finding), symptom or disease temporally associated with the use of the study medication, whether or not considered related to the study medication.

16.2 Adverse Reaction (AR)

All untoward and unintended responses to a medicinal product related to any dose.
The phrase “responses to a medicinal product” means that a causal relationship between a study medication and an AE is at least a reasonable possibility, i.e., the relationship cannot be ruled out.

16.3 Medically significant adverse events

All adverse reactions taking place between Visit 1 and Visit 2 requiring medical consultation with the General Practitioner, Emergency Department, or leading to a subject’s withdrawal (excluding pre-planned visits and GP or Emergency Department

visits for routine medical care) will be considered to be medically significant adverse events.

B cell responses will be determined in participants enrolled at Oxford and, potentially, other study sites according to local arrangements. This analysis will be conducted on fresh or frozen samples at Oxford or other sites. Where applicable, antigen specific B cell responses to vaccines received in the study will be analysed by ELISPOT and flow cytometric assays depending on the available volumes of blood.

16.4 Unexpected Adverse Reaction

An adverse reaction, the nature or severity of which is not consistent with the summary of product characteristics.

16.5 Serious or Severe Adverse Events

To ensure no confusion or misunderstanding of the difference between the terms "serious" and "severe", which are not synonymous, the following note of clarification is provided:

The term "severe" is often used to describe the intensity (severity) of a specific event (as in mild, moderate, or severe myocardial infarction); the event itself, however, may be of relatively minor medical significance (such as severe headache). This is not the same as "serious," which is based on patient/event outcome or action criteria usually associated with events that pose a threat to a patient's life or functioning. Seriousness (not severity) serves as a guide for defining regulatory reporting obligations.

16.6 Serious Adverse Event or Adverse Drug Reaction

A serious adverse event (experience) or reaction is any untoward medical occurrence that at any dose:

- Results in death,
- Is life-threatening,

NOTE: The term “life-threatening” in the definition of “serious” refers to an event in which the patient was at risk of death at the time of the event; it does not refer to an event which hypothetically might have caused death if it were more severe.

- Requires inpatient hospitalisation or prolongation of existing hospitalisation,
- Results in persistent or significant disability/incapacity, or
- Is a congenital anomaly/birth defect.

Medical and scientific judgment should be exercised in deciding whether an adverse event is serious in other situations.

16.7 Expected Adverse Reactions

For all expected adverse reactions see summary of the product characteristics for each vaccine.

16.8 Suspected Unexpected Serious Adverse Reactions (SUSAR)

A serious adverse reaction, the nature or severity of which is not consistent with the applicable product information.

17. CAUSALITY ASSESSMENT

The relationship of medically significant AEs to the study medication will be assessed by a medically qualified investigator according to the following criteria:

-Related: If the causal relationship between the IMP and the SAE is at least a reasonable possibility, i.e., the relationship cannot be ruled out.

-Not related: If there is no causal relationship between the IMP and the SAE i.e. the event is caused by something other than the IMP e.g. underlying disease, a concomitant medication.

18. REPORTING PROCEDURES FOR SERIOUS ADVERSE EVENTS

All Serious Adverse Events (SAEs) must be reported to the CI and sponsor (University of Oxford Clinical Trials and Research Governance Office) within one working day of discovery or notification of the event. All SAE information must be recorded on a SAE form and faxed or a scanned copy emailed to the sponsor.

In addition, SAEs that are experienced by participants taking a Pfizer product must also be reported to Pfizer within one working day of discovery or notification of the event. The SAE must be recorded on the SAE form belonging to the manufacturer to whom it will be faxed or scanned copy emailed.

Additional information received for a case (follow-up or corrections to the original case) need to be detailed on a new SAE form and faxed to the sponsor and to Pfizer

The CI, in conjunction with Pfizer, will report suspected adverse reactions which are both serious and unexpected (SUSARs) experienced by participants taking a Pfizer product to the Competent Authorities (MHRA) and the REC that gave a favourable opinion for the study.

Fatal or life-threatening SUSARs must be reported within 7 days and all other SUSARs within 15 days. In addition to the expedited reporting above, the sponsor/CI shall submit once a year throughout the clinical trial or on request a safety report to the Competent Authority and Ethics Committee.

In addition to the above, the local PI will be responsible for reporting any SUSARs to the relevant NHS Research and Development offices in their area.

The CTRG will ensure that all SAEs are reviewed by medical monitors on a weekly basis and at the next meeting of the University of Oxford Trials Safety Group (TSG), who will meet at regular intervals and consider:

- Occurrence and nature of adverse events
- Whether additional information on adverse events is required
- Consider taking appropriate action where necessary to halt trials
- Act / advise on incidents occurring between meetings that require rapid assessment (e.g. SUSARs).

18.1 Reporting Procedures for Adverse Events

All AEs occurring in the first 4 days after pneumococcal immunisation, and all medically significant adverse events occurring during the study observed by the investigator or reported by the participant, whether or not attributed to study medication, will be reported on the CRF/source document, including the diary card. Reactions occurring in the first 4 days after immunisation will be divided up into solicited and unsolicited reactions.

Adverse events solicited in the diary card that are ongoing after day 4 (as recorded in the diary card provided) will similarly be recorded in the CRF/source document.

The following information will be recorded for medically significant AEs: description, date of onset and end date, severity, assessment of relatedness to study medication, other suspect drug or device and action taken. Follow up information should be provided as necessary.

18.2 Solicited Reactions

This refers to terms/symptoms that are pre-listed in the diary card. Solicited reactions are those that have previously been reported with the use of the study vaccines and, unless stated otherwise, will be considered related to the vaccine.

Solicited reactions will be recorded and monitored by the parent/legal guardian on the diary card provided by the study team from the day of immunization until Day 4 post-immunisation with the PCV13 booster vaccine.

18.2.1 Local Reactions

Local injection site reactions (redness, swelling and tenderness) at the site of PCV13 booster injection will be monitored daily for 4 days (day 0 to day 4) after each vaccination. An end date will be recorded for any reactions persisting after day 4.

18.2.2 Redness and Swelling

Redness and swelling will be measured and recorded on the diary card. They will be categorized as absent, mild, moderate and severe based on the scale given below. A ruler will be given to the parent/legal guardian with instructions for measuring any redness or swelling at the injection site. The parent/legal guardian will be asked to measure the largest diameter of a local reaction and record this in the diary given at visit 1.

Absent	No redness or swelling present (0mm)
Mild	0.5cmm to 2.0 cm
Moderate	2.5 cm to 7.0 cm
Severe	> 7.0 cm

18.2.3 Tenderness

The parent/legal guardian will be asked to assess and record in the diary card whether tenderness is present at the injection site and grade it on the following scale:

0	Absent
1	Tenderness present, does not interfere with routine activities

2	Tenderness present, cries or shows discomfort while doing routine activities
3	Tenderness interfering with limb movement

18.3 General Reactions

18.3.1 Temperature

Axillary temperature will be measured by the parent/legal guardian and recorded from the vaccination day (day 0) to day 4 post-vaccination. A digital thermometer will be provided by the study team on Visit 1. Temperature will be considered as fever if >38.0 centigrades. In the event of fever, temperature will be collected daily until its resolution.

18.4 Unsolicited Reactions

These are any adverse events that are not pre-listed in the diary card but may be reported on the diary card by the participant or through interview with the participant. These will be reported in the CRF/source document adverse event form.

AEs considered related to the study medication by the investigator or the sponsor will be followed until resolution or the event is considered stable.

The following attributes must be assigned by the investigator to all adverse events occurring within 4 days of pneumococcal immunisation and all medically significant adverse events occurring during the study: description, date of onset and resolution date, severity, assessment of relatedness to study medication, other suspected drug or device and action taken.

The investigator may be asked to provide follow-up information.

All related AEs that result in a participant's withdrawal from the study or are present at the end of the study, should be followed up until a satisfactory resolution occurs.

All deaths occurring during the study must be reported to the Sponsor/CI. These include deaths within 30 days of the final dose of study medication and deaths up to the last formal follow-up observational period, whichever is longer. For all deaths, available autopsy reports and relevant medical reports will be made available for reporting to the relevant authorities.

It will be left to the investigator's clinical judgment whether or not an AE is of sufficient severity to require the participant's removal from treatment. A participant may also voluntarily withdraw from treatment due to what he or she perceives as an intolerable AE. If either of these occurs, the patient must undergo an end of study assessment and be given appropriate care under medical supervision until symptoms cease or the condition becomes stable. The severity of events will be assessed on the following scale: 1 = mild, 2 = moderate, 3 = severe. The relationship of AEs to the study medication will be assessed.

18.5 Other reportable information

The following events will also be reported to Pfizer:

- Report of pregnancy exposure to a Pfizer product (information about use in pregnancy encompasses the entire course of pregnancy and delivery, perinatal and neonatal outcomes, even if there were no abnormal findings)
- Report of lactation exposure to a Pfizer product
- Overdose
- Spontaneous reports of an unexpected therapeutic or clinical benefit associated with the use of a Pfizer product
- Abuse (for example, use for non-clinical reasons)
- Inadvertent or accidental exposure
- SIDS
- Apnoea
- Autism
- Hypotonic hyporesponsive episode (HHE)

- Metal poisoning
- Lack of effect

19. STATISTICS AND ANALYSIS

19.1 Number of Participants

Of the 286 children randomised in the original study, approximately 240 were enrolled in the sites that will be participating in this follow on study. Of these, approximately 200 completed the study. It is anticipated that 50% of these participants are likely to take part in this follow-on study, therefore the study population is likely to be approximately 100 children. It is expected that approximately 50% of these will have received PCV13 and the other 50% PCV7.

19.2 Description of statistical methods

Two sided binomial exact confidence intervals will be constructed around the proportion of subjects achieving specified antibody concentrations, and around rates of local and systemic reactions.

The antibody concentrations will be logarithmically transformed for analysis, since the untransformed values are highly skewed. For each serotype separately, and at each time point, geometric means of the antibody concentrations from each of the blood draws will be calculated. Two-sided, 95% confidence intervals will be constructed by back transformation of the confidence intervals for the mean of the logarithmically transformed assay results computed using the Student t distribution.

In addition, the geometric mean fold-rise for the serotype-specific concentrations will be derived from the exponent of the difference between the logarithmically transformed assay results before and after the booster dose. The corresponding 95% confidence intervals will be computed using the same method mentioned above, i.e. the confidence interval will be obtained on the log transformed scale, and then back transformed to give the result on the original scale.

A Chi-square test or Fisher’s exact test will be used to compare the proportions between groups of participants.

Two-sample t-test will be used to compare the logarithmically transformed concentrations between the groups, or else analysis of variance, to adjust for baseline values, as appropriate. Differences between the two groups and the corresponding 95% confidence intervals will be calculated. Comparisons between the two groups will be carried out based on the allocated groups.

A full statistical analysis plan will be prepared by the study statistician prior to the analysis.

19.3 Level of statistical significance

We shall take $p<0.05$ to denote statistical significance, as this is the standard convention.

19.4 Analysis and endpoints

For the primary objective (persistence of antibody to 40 months) the analysis population will consist of all participants who provide a blood sample at visit 1 (intention to treat population for antibody persistence),

For the response to PCV13 immunogenicity analyses, the analysis population will consist of all participants receving a dose of PCV13 and providing a blood sample at visit 2 (intention to treat population for booster dose response).

For analysis of reactogenicity , the analysis population will be defined as all those receiving a dose of PCV13 and providing any reactogenicity or safety data (intention to treat analysis for reactogenicity).

For each of the above analyses, a per-protocol population analysis (excluding those meeting the criteria defined in points 2 a, b or c in section 8.2) will be performed.

For each of the pneumococcal serotypes included in the PCV13 the proportion of subjects achieving an IgG antibody concentration ≥ 0.35 mcg/mL at 40 months of age after the infant series will be computed along with a 2-sided 95% confidence interval for the proportion using the Binomial exact method.

For each serotype of pneumococcal IgG separately, geometric means of the antibody concentrations from each of the blood draws will be calculated, along with their 95% confidence intervals. Similar procedures will be used to evaluate the pre- and post 40 month booster dose assay data. In addition, the geometric mean fold-rise for the serotype-specific pneumococcal IgG concentrations will be derived from the exponent of the difference between the logarithmically transformed assay results before and after the booster dose, with corresponding 95% confidence intervals.

The proportion of participants with antibody concentrations ≥0.35 mcg/mL between those primed with PCV7 and PCV13 will be compared. Differences between the two groups and the corresponding 95% confidence intervals will be calculated.

The rates of reactogenicity of the pre-school PCV13 booster (in terms of rates of each separate local and systemic reactions following vaccination) will be presented along with their 95% confidence intervals.

Reactogenicity data will be summarised descriptively and the association between any genetic polymorphism and outcomes of booster does will be assessed using Fisher's exact test.

B cell response data will be summarised descriptively.

20. DIRECT ACCESS TO SOURCE DATA/DOCUMENTS

Direct access to these will be granted to authorised and trained representatives from the sponsor, host institution and the regulatory authorities to permit trial-related monitoring, audits and inspections.

21. QUALITY CONTROL AND QUALITY ASSURANCE PROCEDURES

The study will be conducted in accordance with the current approved protocol, ICH GCP, relevant regulations and standard operating procedures.

Regular monitoring will be performed according to the ICH GCP. Monitoring of this study will be conducted by freelance monitors in collaboration with the quality assurance manager of the Oxford Vaccine Group and local staff at each study centre.

Following written standard operating procedures and an approved monitoring plan, the monitors will verify that the clinical trial is conducted and data are generated, documented and reported in compliance with the protocol, GCP and the applicable regulatory requirements.

A trial steering committee will be formed that will include, but not be limited to, the chief investigator, a statistician, a quality assurance manager and project manager.

22. ETHICS

22.1 Declaration of Helsinki

The Investigator will ensure that this study is conducted in accordance with the ethical principles of the current revision of the Declaration of Helsinki.

22.2 CH Guidelines for Good Clinical Practice

The Investigator will ensure that this study is conducted in accordance with the applicable regulatory requirements with the principles of the current revision of the ICH Guidelines for Good Clinical Practice.

22.3 Other Ethical Considerations

No study procedures or evaluations will occur until after informed consent is obtained.

The burden of pneumococcal disease is highest among those aged less than five years, and therefore the immunogenicity and antibody persistence of the vaccine needs to be tested in children who are the age group who will receive the booster pre-school vaccine if introduced into routine immunisation.

The nature of the investigational product will be made clear to parents.

23. PARTICIPANT CONFIDENTIALITY

The study staff will ensure that the participants' anonymity is maintained. The participants will be identified only by initials and a participants ID number on the CRF and any electronic database. All documents will be stored securely and only accessible by study staff and authorised personnel. The study will comply with the Data Protection Act which requires data to be anonymised as soon as it is practical to do so. Any data or samples that relate to participants and that leave the study site will be identified by study number and or code only.

24. COMPENSATION FOR HARM

In the very unlikely event that a participant suffered any harm during the duration of the trial, compensation for harm arising from the study would be provided by the vaccines manufacturers.
The sponsor will provide compensation from harm arising from participation in the study that is not due to the study treatment.

25. DATA HANDLING AND RECORD KEEPING

All study files (paper and electronic) with demographic and clinical details on the participants will be kept in a locked research office at each participating study centre. The study details will subsequently be entered on to a computer with an electronic

database protected by a password. All blood samples will be identified by study number only and will have no personal identifiers.

26. FINANCING AND INSURANCE

The study is funded by a grant from Pfizer and will be supported by staff funded by the NIHR Oxford Biomedical Research Centre, Southampton Wellcome Trust Clinical Research Facility, Hampshire and Isle of Wight Comprehensive Local Research Network and Medicines for Children Research Network South-West. Insurance will be provided by the University of Oxford for staff and study participants. The respective vaccine manufacturers will be responsible for product liability.

27. APPENDIX A: STUDY FLOW CHART

Send Recruitment letter and information leaflet to parents/legal guardians of participants who completed the Wyeth-sponsored PCV13 infant trial study (6096A1-007) and agreed to be contacted for this follow on study.

Parents/guardians who reply confirming interest in taking part in the study are telephoned with further information and telephone-screening is carried out to check initial eligibility<br>If eligible, arrange Visit 1

Visit 1: Gain consent<br>Check eligibility<br>Study number allocation (same as for the previous study)<br>Obtain 8-10 ml blood sample<br>Administer PCV13<br>Arrange Visit 2

Visit 2: Check eligibility<br>Obtain 8-10 ml blood sample<br>Administer MMR and Repevax or Infanrix-IPV<br>Complete Study Paperwork

Information entered onto database

Blood transported to laboratory, processed, and serum stored in freezer

Measurement of antibody concentration/OPA/B cell responses

Data analysed

28. REFERENCES

1- http://www.preventpneumo.org/diseases/

2- http://www.who.int/vaccines/en/pneumococcus.shtml Accessed 5th Feb 2009

3- http://www.hpa.org.uk/web/HPAwebFile/HPAweb_C/1245581527892

4- Cardozo et al. Nasopharyngeal Colonization and Penicillin Resistance Among Pneumococcal Strains: A Worldwide 2004 Update. The Brazillian Journal of Infectious Diseases 2006; 10(4):293-303.

5- Whitney CG, Farley MM, Hadler J, et al. Decline in invasive pneumococcus disease after the introduction of protein-polysaccharide conjugate vaccines. N Engl J Med 2003;348:1737-1746

6- Hausdorff WP, Siber G and Paradiso PR. Geographical differences in invasive pneumococcal disease rates and serotype frequency in young children. Lancet 2001;357:950-952

7- WHO position paper: 23-valent pneumococcal polysaccharide vaccine. Weekly epidemiological Record. 2008; 42; 373 384.

8- Black SB, Shinefield HR, et al. Effectiveness of heptavalent pneumococcal conjugate vaccine in children younger than five years of age for prevention of pneumonia. Pediatr Infect Dis J 2002;21:810-815

9- Black SB Shinefield HR et al. Postlicensure surveillance for pneumococcal invasive disease after use of heptavalent pneumococcal conjugate vaccine in Northern California Kaiser Permanente. Pediatr Infect Dis J 2004;23:485-489

10- Direct and Indirect Effects of Routine Vaccination of Children with 7-valent Pneumococcal Conjugate Vaccine on Incidence of Invasive Pneumococcal Disease – United States, 1998-2003. MMWR 2005; 54:893-897.

11- McIntosh ED, Conway P, Willingham J, Hollingsworth R and Lloyd A. Pneumococcal pneumonia in the UK – how herd immunity affects the cost-effectiveness of 7-valent pneumococcal conjugate vaccine (PCV). Vaccine 2005;23:1739-1745.

12- Vaccine Escape Recombinants Emerge after Pneumococcal Vaccination in the United States; AB. Brueggemann, R Pai, D. Crook, B. Beall; PLoS Pathogens 1628 November 2007 | Volume 3 | Issue 11 | e168

13 - http://www.emea.europa.eu/pdfs/human/opinion/Prevenar13 54641709en.pdf

14 - Kaye P, Malkani R, Martin S, et al. Invasive Pneumococcal Disease (IPD) in England & Wales after 7-valent conjugate vaccine (PCV7); potential impact of 10 and 13-valent vaccines. Health Protection Agency, **2009**

15- Infectious Disease Society of America 2008 Poster presentation: Immunogenicity Of DTaP-IPV-Hib And MenC Vaccines In The UK When Administered With a 13-valent Pneumococcal Conjugate Vaccine C. L. Klinger et al.

16- Chapter 15, Functional Assays for Pneumococcal Antibody; Pneumococcal Vaccines; The Impact of Conjugate Vaccines. Edited by G Siber, K Klugman & H Makela. 2008 ASM Press

17 - Blanchard-Rohner G, Pulickal AS, Jol-van der Zijde CM, Snape MD, Pollard AJ. Appearance of peripheral blood plasma cells and memory B-cells in a primary and secondary immune response in humans. Blood. 2009 (epub ahead of print).

18 - D F Kelly, MD Snape, E C Clutterbuck, S Green, C Snowden, L Yu, A Borkowski, E R Moxon and A J Pollard. Conjugated serogroup C meningococcal capsular polysaccharide, but not the native polysaccharide, induces persistent antigen specific memory B cells. Blood. 2006; 108: 2642-7
